# Liverwort and Hornwort Flora of Hoàng Liên National Park and the Adjacent Areas (North Vietnam, Indochina)

**DOI:** 10.3390/plants12091841

**Published:** 2023-04-29

**Authors:** Vadim A. Bakalin, Ksenia G. Klimova, Van Sinh Nguyen, Hung Manh Nguyen, Daniil A. Bakalin, Seung Se Choi

**Affiliations:** 1 Laboratory of Cryptogamic Biota, Botanical Garden-Institute FEB RAS, Makovskogo Street 142, 690024 Vladivostok, Russia; ksenia.g.klimova@mail.ru (K.G.K.); daniil.bakalin@gmail.com (D.A.B.); 2 Institute of Ecology and Biological Resources, Vietnam Academy of Science and Technology, Graduate University of Science and Technology, Ha Noi 10072, Vietnam; nh.manhiebr@gmail.com; 3 Team of National Ecosystem Survey, National Institute of Ecology, Seocheon 33657, Republic of Korea

**Keywords:** Marchantiophyta, Anthocerotophyta, liverworts, hornworts, diversity, distribution patterns, Hoàng Liên Sơn, Vietnam, Indochina

## Abstract

The study of the flora located in the central part of the Hoàng Liên Sơn Range in the northern region of Indochina has revealed 279 species of liverwort and hornwort, 26 of which are newly reported for the flora of Vietnam. The uniqueness and peculiarity of the studied flora are explained by the significant altitudinal range in the area treated and its position in the contact zone of the Sikang-Yunnan floristic province of the East Asian Floristic Region with the Indochina Floristic Region. The checklist includes data on the distribution of each species in the studied region, habitats, and accompanying taxa. The high disunity of the regional floras of the southern tip of the East Asian region compared to the lesser disunity of the regional floras in the north of the East Asian region is shown. In general, the studied flora possess Sino-Himalayan mountain subtropical characteristics with the large participation of tropical elements.

## 1. Introduction

The Hoàng Liên Sơn Range, stretching ca. 180 km, is located in the northern part of Vietnam and is an orographic continuation of the Hengduan Mountains, ranging from the southeastern edge of the Tibet Upland to Indochina. The system of the mountain ranges of Northern Vietnam is a site of penetration of the Sino-Himalayan flora to the south [1,2] and a zone of interpenetration of the mountainous subtropical flora of the Sikang-Yunnan floristic province and the tropical flora developed in the lowlands. The mixing of different floral elements and the significant altitudinal differences (to 3143 m a.s.l.) contribute to the formation of the peculiar and taxonomically rich flora in different taxonomic groups. As Averyanov et al. ([3]: 35) pointed out, “Approximately 15.3% of Vietnamese endemics occur in the Vietnamese portion of the Sikang-Yunnan Province”, and this is only one of the peculiar features of the area. The second noticeable feature worth mentioning here is the great variety of rock compositions in the Hoàng Liên Sơn Range, which vary from acid granites to distinctly alkaline limestone raised up to 1800–2000 m a.s.l. The special phytogeographic importance of the region inside Indochina, including the highest point in Indochina, elicits interest in the study of the Hoàng Liên Sơn Range. The liverworts of the Hoàng Liên Sơn Range were studied fragmentarily, summary data were never published, only scattered floristic findings were disseminated across various papers, and the distribution of species in taxonomic treatments was mentioned [4,5,6,7,8,9,10,11,12,13,14,15,16,17]. Moreover, for Northern Indochina, there are no published summary lists for any local flora at all. Even excluding Laos, where information about liverworts is generally fragmentary [18], the well-studied areas [19] where much work has been carried out—for example, in the Doi Inthanon area—we do not have a summarized taxonomic checklist for any national parks or floristically important areas located in the northern part of the country. The absence of such compendia prevents an adequate understanding of the nature of the liverwort flora in Northern Indochina and its relationships with northwardly adjacent regions of Southwest China.

Considering these facts, we made an attempt to summarize and systematize the data on the liverwort flora of Hoàng Liên National Park and its environs, located in the central part of the Hoàng Liên Sơn Range. To date, all the materials collected by our team from 2016 to 2022 have been identified. The presentation of the data obtained, together with an attempt to analyze the characteristic features of the liverwort flora of the studied area, are the main tasks of the present account.

## 2. Results

### 2.1. Taxonomic Liverwort Diversity in Studied Area

The total number of revealed taxa is 279, belonging to 41 families and 80 genera of Marchantiophyta and two families and two genera of Anthocerotophyta. Among families, the leading position is occupied by Lejeuneaceae, which comprises more than 16% of the flora, followed by Plagiochilaceae and Lepidoziaceae. Among the genera, the most taxonomically rich is *Plagiochila* (almost 11% of the total flora), followed by *Bazzania* and *Lejeunea*. The data on the first 10 leading families and genera are presented in Table 1.

### 2.2. Checklist

Taxa in the checklist are in alphabetical order; nomenclature follows Söderström et al. [20], with the exception of Solenostomataceae, where the narrow genus concept was adopted [21], and for new concepts of taxa in *Calypogeia* and *Chiastocaulon* according to Bakalin et al. [10] and Patzak et al. [22], respectively. Each species is annotated by the following scheme: (1) species name, (2) altitude(s) where the species was collected, (3) brief description of habitat, (4) numerals of collecting localities in accordance with that described in the Section 4.1 of the paper, (5) selected field numbers (by one per locality), (6) accompanying species if any (questionable identifications are omitted), (7) brief citation of literature reports—where present (the spelling of geographic names corresponds to those in cited literature), literature reports by the authors of the present paper are only referenced, but not annotated, and the literature sources are referenced in square brackets, (8) taxonomic comments, if any. The species newly recorded for Vietnam are marked with an asterisk.

*Acrobolbus ciliatus* (Mitt.) Schiffn.—2610, 2846—Partly shaded mesic cliff crevices and open decaying wood—**21**, **24**—V-11-3-17, V-17-33-18—*Plagiochila semidecurrens* [8].

*Acrolejeunea infuscata* (Mitt.) Jian Wang bis et Gradst.—2030, 2100, 2210—Moist to mesic decaying wood, tree trunks, moist cliffs—**12**, **16**, **18**—V-2-46-16, V-4-54-17, V-5-41-17—*Frullania nepalensis*, *Plagiochila subtropica*; Montes Hoang-Lien-Son, in rupibus marmoreis cacuminis supra opp. Sapa, 1780 m, 27 Sept. 1963, T. Pócs 2573/r [23].

*Acrolejeunea recurvata* Gradst.—1565—Mesic tree trunk in part shade in mostly planted forest—**15**—V-11-8-22.

*Acrolejeunea sandvicensis* (Gottsche) Steph.—1565—Mesic tree branches in mostly planted forest—**15**—V-11-12-22—*Frullania obscura*, *F. pocsantha*, *Lejeunea flava*.

*Anastrophyllum assimile* (Mitt.) Steph.—3143—Moist open cliffs—**30**—V-3-38-16 [4].

*Anastrophyllum bidens* (Reinw., Blume et Nees) Steph.—2030, 2610, 2846, 3105, 3143, 3143-3050—Moist to mesic, mostly open cliffs—**21**, **24**, **26**, **27**, **28**, **30** –V-11-1-17, V-17-12-18, V-18-13-18, V-3-17a-16, V-8-21-17—*Bazzania angustistipula*, *B. ovistipula*, *B. praerupta*, *Fuscocephaloziopsis gollanii*, *Herbertus armitanus*, *Metacalypogeia alternifolia*, *Mylia vietnamica*, *Scapania ciliatospinosa*, *S. ornithopoides*, *Schistochilopsis setosa*, *Syzygiella nipponica* [4].

*Aneura maxima* (Schiffn.) Steph.—3143—Moist cliffs in part shade—**30**—V-3-21-16 [4].

*Aneura pinguis* (L.) Dumort.—1557, 1620, 1900, 2610, 2884, 2900—Moist cliffs and boulders, including those near watercourses in part shade—**5**, **6**, **8**, **21**, **23**, **23**—V-1-110-16, V-11-8-17, V-13-5-17, V-20-9-18, V-8-36-22, V-9-21-17—*Bazzania ovistipula*, *Herbertus armitanus*, *Mnioloma fuscum*, *Mylia vietnamica*, *Plagiochila semidecurrens*, *Preissia quadrata*, *Scapania ferruginea*, *S. ornithopoides* [4].

*Anthoceros angustus* Steph.—1557—Open moist clayish trail side—**5**—V-8-23-22.

*Asperifolia indosinica* Bakalin et A.V. Troitsky—1557, 1520, 1900, 2100, 2670—Moist fine soil covering rocks and stream banks, in partly shaded to open habitats—**5**, **8**, **12**, **22**—V-1-112-16, V-2-22-16, V-8-44-22, V-19-10-18, V-3-14-17—*Calypogeia cuspidata*, *Riccardia pumila*, *Cephalozia siamensis* [10].

*Asterella cruciata* (Steph.) Horik.—2014—Open moist humus covering cliffs near waterfall—**11—**V-21-3-18.

*Asterella wallichiana* (Lehm. et Lindenb.) Grolle—1860, 1900—Mesic cliffs and moist clayish road cuts—**4**, **8**—V-1-80-16, V-10a-1-17 [4].

*Bazzania angustifolia* Horik.—Phan-Si-pan, 2100 m a.s.l. [15]; Sapa, 1725 m a.s.l. [13,16].

*Bazzania angustistipula* N. Kitag.—1840, 2030, 2060, 2210, 2846, 2884, 2900, 2957, 3105—Open to partly shaded mesic to moist cliffs, tree trunks and branches, decaying wood—**8**, **13**, **16**, **18**, **23**, **24**, **25**, **26**, **27**, **29**—V-10-27-17, V-17-8-18, V-18-2-18, V-20-5-18, V-4-49-17, V-5-43-17, V-6-41-17, V-8-10-17, V-9-10-22—*Anastrophyllum bidens*, *Bazzania ovistipula*, *Calypogeia granulata*, *Drepanolejeunea angustifolia*, *Herbertus dicranus*, *Heteroscyphus tener*, *Kurzia makinoana*, *Lejeunea flava*, *Lepidozia subtransversa*, *Leucolejeunea turgida*, *Metacalypogeia alternifolia*, *Metzgeria leptoneura*, *Plagiochila beddomei*, *P. gracilis*, *P. pseudofirma*, *P. sciophila*, *P. trabeculata*, *Pleurozia subinflata*, *Radula cavifolia*, *Scapania ciliatospinosa*, *S. ornithopoides*.

*Bazzania faurieana* (Steph.) S. Hatt.—Sapa, 2000 m a.s.l. [13,16].

*Bazzania himalayana* (Mitt.) Schiffn.—1900, 2100 2060, 2210—Tree trunks, decaying stumps and mesic cliffs in part shade—**8**, **12**, **13**, **16—**V-1-39-16, V-2-115-16, V-5-51-17, V-6-40-17—*Leucolejeunea turgida*, *Plagiochila semidecurrens* [4].

*Bazzania japonica* (Sande Lac.) Lindb.—1410, 1520, 1557, 1840, 1995, 2014, 2030, 2210—Open to partly shaded moist to mesic cliffs, including those near streams, rarely open tree trunks—**3**, **5**, **5**, **8**, **10**, **11**, **16**, **18**—V-10-21-17, V-2-5-17, V-21-12a-18, V-3-44-17, V-4-60-17, V-5-56-17, V-7-6-17, V-8-15-22—*Bazzania ovistipula*, *B. tridens*, *Calypogeia granulata*, *Heteroscyphus coalitus*, *Lepidozia faurieana*, *L. subtransversa*, *Metacalypogeia alternifolia*, *Plagiochila assamica*, *Riccardia decrescens*, *Scapania maxima*, *Trichocolea rudimentaris*.

*Bazzania aff. *mayebarae* S. Hatt.—1557—Boulders and humus covering rocks in part shade—**5—**V-8-26-22—*Calypogeia tosana*. Comment: *Bazzania mayebarae* is distinctive due to distant, bilobed leaves; the hyaline underleaves are appressed to the stem and 1.5–2.2 times wider than the stem, with a distinctly verruculose leaf cuticle. *Bazzania debilis*, described from Thailand, in contrast to *B. mayebarae*, described from Japan, is very similar to the latter, although different in clearly bilobed leaves and longer than wide underleaves (versus leaves unclearly bilobed and underleaves wider than long). The Vietnamese plants commonly have clearly bilobed leaves (feature of *B. debilis*), wider than long underleaves (feature of *B. mayebarae*) and ovate leaves (feature of *B. mayebarae*, while *B. debilis* has oblong ovate to sublinear leaves). In fact, we estimate that these two names may be synonymous and taxonomical relationships within this pair should be further investigated.

*Bazzania oshimensis* (Steph.) Horik.—1557—Open mesic cliffs near stream—**5**—V-8-16-22; as *Bazzania tridens* var. *oshimensis* (Steph.) Pócs: Sapa, 1600 m a.s.l. [16].

*Bazzania ovistipula* (Steph.) Abeyw.—1840, 2030, 2060, 2210, 2610, 2727, 2835, 2846, 2884, 2900, 2957, 3105—Open, rarely partly shaded, cliffs including those near streams, rarely moist decaying wood in part shade—**8**, **13**, **16**, **18**, **17**, **18**, **19**, **21**, **23**, **23**, **24**, **26**, **27**, **29**, **31**—V-10-17-22, V-11-13-17, V-12-19-17, V-15-14-18, V-17-2-18, V-18-14-18, V-20-7-18, V-4-10-17, V-5-30-17, V-6-17-17, V-8-30a-17, V-9-29-17—*Anastrophyllum bidens*, *Aneura pinguis*, *Bazzania angustistipula*, *B. japonica*, *B. tridens*, *Calycularia crispula*, *Calypogeia tosana*, *Cephaloziella willisana*, *Cololejeunea schmidtii*, *Delavayella serrata*, *Frullania davurica*, *F. moniliata*, *F. nepalensis*, *Fuscocephaloziopsis gollanii*, *Herbertus armitanus*, *Heteroscyphus argutus*, *H. coalitus*, *H. tener*, *Kurzia makinoana*, *Lepidozia apiculata*, *L. omeiensis*, *Marsupella vietnamica*, *Metacalypogeia alternifolia*, *Mylia vietnamica*, *Plagiochila beddomei*, *P. gracilis*, *P. semidecurrens*, *P. trabeculata*, *Radula cavifolia*, *Scapania contorta*, *S. ciliatospinosa*, *S. metahimalayana*, *Schistochilopsis setosa*, *Solenostoma* cf. *pseudocyclops*, *Sphenolobopsis pearsonii*, *Spruceanthus semirepandus* [4]; Phan-si-pan, 2000–3000 m a.s.l..

*Bazzania pearsonii* Steph.—1900, 2060, 2100, 2210, 2670, 2727, 3143—Open to partly shaded, moist to mesic fallen decaying tree trunks, rarer mesic cliffs and living tree trunk bases—**8**, **12**, **13**, **16**, **19**, **22**, **30**—V-1-68-16, V-2-67-16, V-3-51-16, V-15-16-18, V-19-8-18, V-5-78c-17, V-6-34-17—*Bazzania tridens*, *Delavayella serrata*, *Heteroscyphus tener*, *Plagiochila semidecurrens*, *Plicanthus birmensis*, *Radula madagascariensis* [4].

*Bazzania praerupta* (Reinw., Blume et Nees) Trevis.—2030, 2210, 2610, 2670, 2727, 2821, 2835, 2846, 2900, 2957, 3105—Open to partly shaded tree trunks, moist cliffs—**16**, **19**, **20**, **21**, **22**, **23**, **24**, **24**, **26**, **27**, **29**, **31—**V-10-24-22, V-11-4-17, V-15-36-18, V-16-10-18, V-17-12-18, V-18-13-18, V-19-35-18, V-5-2-17, V-8-16-17, V-9-15-17—*Anastrophyllum bidens*, *Cephaloziella willisana*, *Cryptolophocolea sikkimensis*, *Delavayella serrata*, *Herbertus ramosus*, *Heteroscyphus tener*, *Metacalypogeia alternifolia*, *Mnioloma fuscum*, *Plagiochila hyalodermica*, *P. semidecurrens*, *Pleurozia gigantea*, *Plicanthus birmensis*, *Scapania ferruginea*, *S. ornithopoides*, *Schistochilopsis setosa*.

*Bazzania recurvolimbata* (Steph.) N. Kitag.—Sapa, 1500 m a.s.l. [16].

*Bazzania tridens* (Reinw., Blume et Nees) Trevis.—1325, 1520, 1840, 2030, 2060, 2210, 2610, 2670, 2727—Open to partly shaded moist to mesic cliffs and boulders, including those near streams, decaying wood, tree trunks, open roots on steep slopes; virtually the most eurytopic species in the flora—**1**, **5**, **8**, **13**, **16**, **18**, **17**, **18**, **19**, **19**, **22**—V-1-28-17, V-10-18-17, V-12-18-17, V-15-24-18, V-19-20-18, V-3-25-17, V-4-32-17, V-5-26-17, V-6-37-17—*Bazzania japonica*, *B. ovistipula*, *B. pearsonii*, *Calypogeia granulata*, *Cephalozia siamensis*, *Delavayella serrata*, *Fuscocephaloziopsis gollanii*, *Heteroscyphus argutus*, *H. coalitus*, *H. tener*, *Lepidozia faurieana*, *Plagiochila hyalodermica*, *P. semidecurrens*, *P. trabeculata*, *Plectocolea granulata*, *Radula javanica*, *Riccardia decrescens*, *Scapania maxima*, *Schistochila doriae*; as *Bazzania oblonga*: Phan-si-pan, 2400 m a.s.l. [15]; Phan-si-pan, 2000–3000 m a.s.l. [16]; as *Bazzania intermedia* (Gottsche et Lindenb.) Trevis.: Sapa [13]; [15].

*Bazzania uncigera* (Reinw., Blume et Nees) Trevis.—1900, 2100—Partly shaded mesic humus on steep slope, tree trunks—**8**, **12**—V-1-20-16, V-2-40-16 [4].

*Bazzania yakushimensis* Horik.—Sapa, 2000 m a.s.l. [13].

*Blepharostoma minus* Horik.—1325—Mesic cliffs in part shade—**1**—V-1-27-17. Comment: this report is published in [24]; other reports are unclear.

*Calycularia crispula* Mitt.—1520, 2030, 2100, 2610, 2846, 2884, 2900, 2957—Partly shaded to open moist to mesic cliffs, rarely decaying wood—**5**, **12**, **17**, **18**, **23**, **23**, **24**, **29**—V-12-10-17, V-17-35-18, V-2-68-16, V-20-7-18, V-3-11-17, V-4-47-17, V-9-14-22—*Bazzania ovistipula*, *Calypogeia aeruginosa*, *C. cuspidata*, *C. granulata*, *Cephaloziella willisana*, *Fuscocephaloziopsis gollanii*, *Metacalypogeia alternifolia*, *Plagiochila hyalodermica*, *P. trabeculata*, *Scapania metahimalayana*, *Solenostoma faurieanum* [4].

*Calypogeia aeruginosa* Mitt.—2030, 2610, 2846, 2957—Moist to wet, mostly open cliffs—**21**, **24**, **26**, **29**—V-11-12-17, V-17-14-18, V-8-24-17, V-9-54-22—*Calycularia crispula*, *Cryptolophocolea sikkimensis*, *Herbertus ramosus*, *Lepidozia faurieana*, *Metacalypogeia alternifolia*, *Plagiochila hyalodermica*, *Scapania ciliatospinosa*, *S. contorta*, *S. ferruginea*, *S. metahimalayana*, *Solenostoma faurieanum*, *S. suborbiculatum* [8].

*Calypogeia apiculata* (Steph.) Steph.—1325, 1410, 1520, 1840, 2210, 2670—Open to partly shaded cliffs and boulders, mostly near streams—**1**, **3**, **3**, **5**, **8**, **16**, **22**—V-1-34-17, V-10-28-17, V-19-27-18, V-2-11-17, V-3-14-17, V-5-16-17—*Asperifolia indosinica*, *Heteroscyphus argutus*, *H. coalitus*, *H. zollingeri*, *Jubula sikkimensis*, *Lepidozia vitrea*, *Plectocolea tetragona*, *Riccardia parvula*, *Scapania parvitexta* [10].

*Calypogeia cuspidata* (Steph.) Steph.—1557, 2835, 2957—Open to partly shaded, moist to wet cliffs and boulders near streams—**5**, **29**, **31**—V-10-32-22, V-8-12-22, V-9-39-22—*Asperifolia indosinica*, *Calycularia crispula*, *Jubula javanica*, *Plagiochila trabeculata*, *Riccardia pumila*, *Solenostoma rotundatum*, *S. suborbiculatum*. Comment: as we showed before [10], the identity of the specimens named *C. cuspidata* from Vietnam with “true” *C. cuspidata* described from Hawaii is not evident.

*Calypogeia granulata* Inoue—1410, 1520, 1840, 1900, 2030, 2060, 2100, 2210, 2610, 2957—Open to partly shaded moist to mesic cliffs near streams and on steep slopes, rarely sandy stream banks—**3**, **5**, **8**, **8**, **12**, **13**, **16**, **17**, **18**, **26**, **29**—V-1-131-16, V-10-13-17, V-12-5-17, V-2-10-17, V-3-83-17, V-4-1-17, V-5-13-17, V-6-28-17, V-8-60-17, V-9-11-22—*Bazzania angustistipula*, *B. japonica*, *B. tridens*, *Calycularia crispula*, *Calypogeia tosana*, *Delavayella serrata*, *Fuscocephaloziopsis catenulata* subsp. *nipponica*, *F. gollanii*, *Heteroscyphus argutus*, *H. coalitus*, *Isotachis japonica*, *Jackiella javanica*, *Lepidozia faurieana*, *L. subtransversa*, *Metacalypogeia alternifolia*, *Plagiochila hyalodermica*, *P. sciophila*, *P. trabeculata*, *Plectocolea granulata*, *P. rosulans*, *Riccardia parvula*, *Scapania ciliatospinosa*, *S. ligulata*, *S. maxima*, *S. ornithopoides*, *Schiffneria hyalina* [4].

*Calypogeia japonica* Steph.—1325, 1350—Open moist cliffs near stream and mesic clay on the edge of rice field—**1**, **2**—V-4-22-16, V-1-31-17 [4].

*Calypogeia lunata* Mitt.—1520, 2014, 2210, 2727, 2821, 2846, 3105—Open, rarely partly shaded, moist cliffs, including those near streams, once on moist decaying wood in part shade—**5**, **11**, **16**, **19**, **20**, **24**, **27**—V-15-9-18, V-16-11-18, V-17-50-18, V-18-16-18, V-21-18-18, V-3-82-17, V-5-24-17—*Cephalozia siamensis*, *Fuscocephaloziopsis gollanii*, *Heteroscyphus coalitus*, *Isotachis japonica*, *Lepidozia faurieana*, *L. omeiensis*, *Saccogynidium muricellum*, *Scapania ornithopoides*.

*Calypogeia pseudocuspidata* Bakalin, Frank Müll. et A.V. Troitsky—2014, 2957—Moist cliff in part shade—**11**, **29**—V-21-17-18 (holotype), V-9-40-22 [10].

*Calypogeia sinensis* Bakalin et Buczk.—2014, 3105—Open moist cliffs near stream—**11**, **27**—V-18-17-18, V-21-23-18—*Delavayella serrata*, *Lepidozia omeiensis*, *Plagiochila hyalodermica*, *Scapania maxima* [10].

*Calypogeia tosana* (Steph.) Steph.—1520, 1557, 1900, 2030, 2100, 2670—Open to partly shaded mesic to moist clayish soil and humus, including those covering rocks and near streams—**5**, **8**, **12**, **22**, **26**—V-1-16-16, V-2-90-16, V-19-11-18, V-3-73-17, V-8-26-22—*Bazzania* aff. *mayebarae*, *B. ovistipula*, *Calypogeia granulata*, *Sphenolobopsis pearsonii* [4].

*Calypogeia vietnamica* Bakalin et Vilnet—2030, 2900, 2957, 3050, 3105—Moist cliffs in part shade—**25**, **26**, **26**, **27**, **29**—V-18-18-18, V-8-48-17, V-9-23-17 (holotype)—*Plagiochila semidecurrens*, *Scapania ciliatospinosa*, *S. ornithopoides* [6].

*Cephalozia albula* Steph.—1557, 2014, 2670—Open moist cliffs and boulders near streams—**5**, **11**, **22**—V-19-2-18, V-21-10-18, V-8-19-22—*Jackiella javanica* [8].

*Cephalozia conchata* (Grolle et Váňa) Váňa—1900, 1995, 2835—Open, rarely partly shaded, moist to wet cliffs near streams—**5**, **8**, **10**, **31**—V-1-125-16, V-10-27-22, V-7-10-17—*Isotachis japonica*, *Lepidozia omeiensis*, *Marsupella vietnamica*, *Riccardia nagasakiensis*, *R. pumila*, *Solenostoma faurieanum* [4].

*Cephalozia hamatiloba* Steph.—1557, 2030, 2821, 3143—Open, rarely partly shaded, moist to wet cliffs—**5**, **20**, **26**, **30**—V-16-1-18, V-3-65-16, V-8-18-22 [4].

*Cephalozia siamensis* N. Kitag.—1410, 1520, 1557, 1840, 1900, 2014, 2030, 2210, 2670—Open to partly shaded moist to wet fine soil, cliffs and boulders near streams, rarely on partly shaded decaying wood—**3**, **5**, **5**, **8**, **8**, **11**, **16**, **18**, **22**—V-1-12-16, V-10-20-17, V-19-10-18, V-2-22-17, V-21-9-18, V-3-50-17, V-4-44-17, V-5-62-17, V-8-2-22—*Asperifolia indosinica*, *Bazzania tridens*, *Calypogeia lunata*, *Isotachis japonica*, *Jackiella javanica*, *Lepidozia faurieana* [4].

* *Cephaloziella willisana* (Steph.) N. Kitag.—2030, 2060, 2100, 2846, 2957—Open to partly shaded moist cliffs—**12**, **24**, **29**, **18**, **13**—V-2-137-16, V-17-35-18, V-9-24-22, V-4-2-17, V-6-4-17—*Bazzania ovistipula*, *B. praerupta*, *Calycularia crispula*, *Herbertus ramosus*, *Plagiochila hyalodermica*, *Scapania ciliatospinosa*. Comment: we do not support the synonymy of *C. willisana* with *Cylindrocolea kiaeri* following [25].

* *Cephaloziopsis exigua* (Inoue) R.M. Schust. et Inoue—1410—Open moist cliff crevice—**3—**V-2-18-17.

*Cheilolejeunea imbricata* (Nees) S. Hatt.—1410—Open mesic branch of living tree—**3**—V-2-12-17—Plagiochila peculiaris, Porella caespitans, Ptychanthus striatus.

*Cheilolejeunea subopaca* (Mitt.) Mizut.—2210—Open mesic tree trunk—**16**—V-5-37-17 [8].

*Chiastocaulon fimbriatum* (Mitt.) S.D.F. Patzak, M.A.M. Renner, Schäf.-Verw. et Heinrichs—1840, 2060—Open mesic tree trunks and partly shaded moist decaying wood—**8**, **13**—V-10-43-17, V-6-32-17—*Drepanolejeunea angustifolia*, *Heteroscyphus tener*, *Leucolejeunea turgida*, *L. soae*, *L. subfusca*, *Plagiochila semidecurrens*, *Plicanthus birmensis*, *Syzygiella elongella*—as *Plagiochilion fimbriatum* (Mitt.) Inoue [8].

*Chiastocaulon mayebarae* (S. Hatt.) S.D.F. Patzak, M.A.M. Renner, Schäf.-Verw. et Heinrichs—2670, 2846—Open mesic cliffs and partly shaded mesic tree trunk—**22**, **24**—V-17-22-18, V-19-19-18—as *Plagiochilion mayebarae* S. Hatt. [8].

*Chiastocaulon theriotianum* (Steph.) S.D.F. Patzak, M.A.M. Renner, Schäf.-Verw. et Heinrichs—1840, 2060—Wet cliffs in full sun near stream—**8**, **13**—V-6-4-17, V-10-35-17, V-10-36-17—*Cylindrocolea recurvifolia*, *Marsupella vietnamica*—as *Plagiochilion theriotianum* (Steph.) Inoue [8].

*Chiloscyphus polyanthos* (L.) Corda—1325—Wet cliff in part shade—**1**—V-1-40-17 [8].

*Cololejeunea appressa* (A. Evans) Benedix—1325—Mesic trunk of living tree in part shade—**1**—V-1-10-17—*Cololejeunea hasskarliana*.

*Cololejeunea denticulata* (Horik.) S. Hatt.—1520, 2100—Partly shaded upper surfaces of the leaves of evergreen shrubs—V-2-27-16, V-3-29-17—**5**, **12**—*Drepanolejeunea angustifolia*, *D. commutata* [4].

*Cololejeunea dozyana* (Sande Lac.) Schiffn.—2100—Partly shaded thin branch of shrub—**12**—V-2-20-16 [4].

*Cololejeunea haskarliana* (Lehm.) Schiffn.—1325, 2030—Mesic tree trunks and moist cliff crevices in part shade—**1**, **18**—V-1-10-17, V-4-37-17—*Cololejeunea appressa*, *Plagiochila fruticosa*.

*Cololejeunea indica* Pandé et R.N. Misra—as *Cololejeunea* cf. *planiflora* Benedix: Phan-si-pan, 2400 m a.s.l. [15].

*Cololejeunea peraffinis* (Schiffn.) Schiffn.—Phan-si-pan, 2400 m a.s.l. [15].

Cololejeunea schmidtii Steph.—2030—Open to partly shaded moist cliffs—**26—**V-8-59-17—Bazzania ovistipula, Delavayella serrata, Scapania ciliatospinosa.

*Cololejeunea yakusimensis* (S. Hatt.) Mizut.—1325—Evergreen shrub leaf upper surface, in part shade—**1**—V-1-7-17—*Drepanolejeunea commutata*.

*Conocephalum japonicum* (Thunb.) Grolle—1325, 1350, 1557, 1900—Partly shaded wet cliffs near watercourses, open clayish roadsides and rice field edges—**1**, **2**, **5**, **8**—V-1-137-16, V-4-16-16, V-8-31-22—*Wiesnerella denudata* [4].

*Conocephalum salebrosum* Szweyk., Buczk. et Odrzyk.—1350—Partly shaded mesic limestone crevice—**2—**V-4-8-16 [4].

*Cryptolophocolea sikkimensis* (Steph.) Bakalin et Maltseva—2030, 2610, 2821, 2835, 2900, 2957, 3143—Open to partly shaded moist cliffs—**17**, **20**, **23**, **26**, **29**, **30**, **31**—V-10-21-22, V-12-17-17, V-16-6-18, V-3-61-16, V-8-13-17, V-9-22-17—*Bazzania praerupta*, *Calypogeia aeruginosa*, *Fuscocephaloziopsis catenulata* subsp. *nipponica*, *Herbertus ramosus*, *Metacalypogeia alternifolia*, *Plagiochila hyalodermica*, *P. semidecurrens*, *Scapania ciliatospinosa*, *S. ferruginea*, *Schistochilopsis setosa* [4,11].

*Cyathodium cavernarum* Kunze—1410—Partly shaded moist crevice between boulders near stream—**3—**V-2-1-17 [26].

*Cylindrocolea recurvifolia* (Steph.) Inoue—1520, 1557, 1840, 1995, 2014, 2030, 2100, 2610, 2846, 2957, 3105—Open, rarely partly shaded, wet to moist cliffs, including those near streams—**5**, **8**, **10**, **11**, **12**, **18**, **21**, **24**, **27**, **29**—V-2-109-16, V-10-32-17, V-11-9-17, V-17-30-18, V-18-12-18, V-21-1-18, V-3-70-17, V-4-42-17, V-7-15-17, V-8-37-22, V-9-48-22—*Chiastocaulon theriotianum*, *Marsupella vietnamica*, *Syzygiella elongella* [4].

*Delavayella serrata* Steph.—2030, 2060, 2100, 2210, 2610, 2727, 2821, 3105, 3143—Open to partly shaded, moist cliffs and decaying wood mostly near streams—**12**, **13**, **16**, **17**, **19**, **20**, **21**, **26**, **27**, **30**—V-2-81-16, V-3-75-16, V-11-7-17, V-12-11-17, V-15-25-18, V-16-10-18, V-18-17-18, V-5-18-17, V-6-37-17, V-8-58-17—*Bazzania ovistipula*, *B. pearsonii*, *B. praerupta*, *B. tridens*, *Calypogeia granulata*, *Cololejeunea schmidtii*, *Fuscocephaloziopsis gollanii*, *Heteroscyphus argutus*, *H. coalitus*, *H. tener*, *Lepidozia omeiensis*, *Metacalypogeia alternifolia*, *Nowellia curvifolia*, *Plagiochila hyalodermica*, *P. semidecurrens*, *Riccardia palmata*, *Scapania ciliatospinosa*, *S. ornithopoides* [4].

*Diplophyllum albicans* (L.) Dumort.—2900—Moist cliff in part shade—**23**—V-9-26-17—*Diplophyllum trollii* [8].

*Diplophyllum nanum* Herzog—1900—Partly shaded fine soil of a trail cut and soil-filled crevices in mesic cliffs—**8—**V-1-5-16 [4].

* *Diplophyllum taxifolium* (Wahlenb.) Dumort.—2846—Partly shaded mesic cliff crevice—**24**—V-17-29-18—*Herbertus armitanus*.

* *Diplophyllum trollii* Grolle—2900—Moist cliff in part shade—**23**—V-9-26-17—*Diplophyllum albicans*.

*Drepanolejeunea angustifolia* (Mitt.) Grolle—1520, 1840, 2030, 2060—Open moist cliffs near streams, partly shaded tree trunks, branches, upper surfaces of leaves, rarely decaying wood—**5**, **8**, **13**, **18**—V-10-27-17, V-3-29-17, V-4-28-17, V-6-14-17– *Bazzania angustistipula*, *Chiastocaulon fimbriatum*, *Cololejeunea denticulata*, *Drepanolejeunea commutata*, *Heteroscyphus tener*, *Lejeunea flava*, *L. neelgherriana*, *Leucolejeunea turgida*, *Lopholejeunea soae*, *L. subfusca*, *Microlejeunea punctiformis*, *Plagiochila beddomei*, *P. semidecurrens*, *Plicanthus birmensis*, *Radula cavifolia*, *R. javanica*, *Syzygiella elongella*; as *Drepanolejeunea tenuis* (Nees) J.B. Jack et Steph.: Phan-si-pan, 2400 m a.s.l. [15].

*Drepanolejeunea commutata* Grolle et R.L. Zhu—1325, 1520—Partly shaded upper surface of the leaf of evergreen shrub—**1**, **5**—V-1-7-17, V-3-29-17– *Cololejeunea denticulata*, *C. yakusimensis*, *Drepanolejeunea angustifolia*.

*Drepanolejeunea herzogii* R.L. Zhu et M. L. So—1900, 2100, 2670—Open mesic trunk of living tree, partly shaded leaf surface and thin branches of shrubs—**8**, **12**, **22**—V-1-33-16, V-2-18b-16, V-19-25-18 [4].

*Drepanolejeunea ternatensis* (Gottsche) Steph.—1410, 2030—Open to partly shaded mesic tree trunks and branches—**3**, **18**—V-2-13-17, V-4-70-17—*Herbertus dicranus*, *Lejeunea flava*, *L. tuberculosa*, *Plagiochila pulcherrima*, *Radula cavifolia*.

*Dumortiera hirsuta* (Sw.) Nees—1325, 1350, 1520, 1900—Mesic to wet cliffs, including those of limestone rocks, mostly near streams—**1**, **2**, **5**, **8**—V-1-37-17, V-3-61-17, V-4-9-16—*Pellia endiviifolia*.

*Frullania apiculata* (Reinw., Blume et Nees) Dumort.—1840, 1900–2100, 2060—Open to partly shaded tree trunks and branches—**8**, **12**, **13**—V-10-52-17, V-2-38-16, V-6-7-17—*Frullania nepalensis*.

*Frullania ericoides* (Nees) Mont.—as *Frullania squarrosa* (Nees ex Mont.) Nees, Sapa, 1780 m a.s.l. [13].

*Frullania meyeniana* Lindenb.—Sapa, 1780 m a.s.l. [13].

*Frullania moniliata* (Reinw., Blume et Nees) Mont.—1565, 1840—Open to partly shaded cliffs, including those made of limestone and near streams—**8**, **15**—V-10-4-17, V-11-20-22—*Bazzania ovistipula*, *Plagiochila beddomei*; Sapa, 1600 m a.s.l. [13]; as *Frullania iwatsukii* subsp. *vietnamica* S. Hatt.: “Leg. polonicus.”, Vietnam, Montes Hoang-Lien-Son, 1650 m a.s.l. [27].

*Frullania motoyana* Steph.—1620, 2900—Open dry cliffs and mesic tree trunks—**6**, **23**—V-13-13-17, V-9-3-17—*Frullania pocsantha*, *Herbertus dicranus*.

*Frullania nepalensis* (Spreng.) Lehm. et Lindenb.—1410, 1520, 1565, 1620, 1700-1900, 1900-2100, 2014, 2030, 2060, 2210, 2610, 2670—Open to partly shaded tree trunks and branches—**3**, **5**, **6**, **8**, **11**, **12**, **13**, **15**, **16**, **17**, **18**, **22**—V-1-37-16, V-11-13-22, V-12-3-17, V-13-17-17, V-19-34-18, V-2-14-17, V-2-38-16, V-21-20-18, V-3-56-17, V-4-55-17, V-5-33-17, V-6-15-17—*Acrolejeunea infuscata*, *Bazzania ovistipula*, *Frullania apiculata*, *Plagiochila pulcherrima*, *P. semidecurrens*, *Plicanthus birmensis*; as *Frullania nishiyamensis* Steph.: Sapa [13]; Sapa, 1600, 1780, 1900 m a.s.l. [13].

*Frullania obscura* (Sw.) Dumort.—1565—Mesic tree branches in part shade—**15**—V-11-11-22—*Acrolejeunea sandvicensis*, *Frullania pocsantha*, *Lejeunea flava*; as *Frullania wallichiana* Mitt.: 1730 m a.s.l. [13].

*Frullania pocsantha* Thaithong et S. Hatt.—1565, 1620—Open to partly shaded mesic tree branches—**6**, **15**—V-11-15-22, V-13-10-17 (our collections may be topotypes)—*Acrolejeunea sandvicensis*, *Frullania motoyana*, *F. obscura*, *Lejeunea flava*; Petelot 32, 1927/1929, Vietnam, Montes Hoang-Lien-Son, prope opp. Sapa, prov. Lao-cai, 1600–1800 m (Holotype: NICH) [27]; as *Frullania physantha* Mitt.: Sapa [13], Sapa, 1650 m a.s.l. [15].

*Frullania* cf. *ramuligera* (Nees) Mont.—Sapa, 2000 m a.s.l. [13].

*Frullania serrata* Gottsche—1700–1900, 1840—Mesic open tree trunks and branches—**8—**V-1-41-16, V-10-15-17.

* *Frullania tagawana* (S. Hatt. et Thaithong) S. Hatt.—2835—Mesic *Rhododendron* branch in part shade—**31**—V-10-15a-22– *Radula cavifolia*, *Vietnamiella epiphytica*.

*Frullania yuennanensis* Steph.—1620, 1840, 1900, 2060, 2100, 2210, 2610—Open, rarely partly shaded tree trunks and branches, including those on fallen decaying trunks—**6**, **8**, **12**, **13**, **16**, **17**—V-1-54-16, V-2-16-16, V-10-14-17, V-12-2-17, V-13-16-17, V-5-38-17, V-6-19-17—*Plagiochila semidecurrens*, *P. subtropica*, *Ptychanthus striatus*, *Radula madagascariensis* [4].

*Fuscocephaloziopsis catenulata* subsp. *nipponica* (S. Hatt.) Váňa et L. Söderstr.—2030, 2060, 2100, 3143—Partly shaded moist cliffs, humus on steep slope and decaying wood—**12**, **13**, **18**, **30**—V-2-94-16, V-3-91-16, V-4-25-17, V-6-23-17—*Calypogeia granulata*, *Cryptolophocolea sikkimensis*, *Heteroscyphus argutus*, *Schiffneria hyalina*, *Schistochilopsis setosa* [4].

*Fuscocephaloziopsis gollanii* (Steph.) Váňa et L. Söderstr.—1557, 2957, 1520, 2014, 2030, 2610, 2670, 2727, 2846, 2900, 2957, 3143—Open to partly shaded moist, rarely wet, cliffs and boulders including those near streams, humus covering rocks—**5**, **11**, **17**, **19**, **22**, **23**, **24**, **26**, **29**, **30**—V-3-73-16, V-8-10-22, V-9-11-22, V-12-12-17, V-15-1-18, V-17-2-18, V-19-15-18, V-21-18-18, V-3-45-17, V-3-73-16, V-8-21-17, V-9-15-22, V-9-36-17—*Anastrophyllum bidens*, *Bazzania ovistipula*, *B. tridens*, *Calycularia crispula*, *Calypogeia granulata*, *C. lunata*, *Delavayella serrata*, *Heteroscyphus coalitus*, *Jubula javanica*, *Kurzia borneensis*, *K. makinoana*, *Metacalypogeia alternifolia*, *Metzgeria leptoneura*, *Plagiochila hyalodermica*, *P. sciophila*, *Radula cavifolia*, *Scapania ornithopoides*, *Schiffneria hyalina*, *Schistochilopsis setosa* [4].

*Gymnomitrion rubidum* (Mitt.) Váňa, Crand.-Stotl. et Stotler—2670—Open mesic stump in scattered *Abies delavayi* forest—**22**—V-19-6-18 [9].

*Haplomitrium mnioides* (Lindb.) R.M. Schust.—1520, 1557—Open wet cliffs and partly shaded moist cliff cave near streams—**5—**V-3-16-17, V-8-35-22.

*Herbertus armitanus* (Steph.) H.A. Mill.—2030, 2610, 2670, 2727, 2821, 2835, 2846, 2884, 2900, 3143—Mostly open, rarely partly shaded mesic to moist cliffs, tree trunk and branches—**18**, **19**, **20**, **21**, **22**, **23**, **24**, **26**, **30**, **31—**V-10-22-22, V-11-8-17, V-15-13-18, V-16-27-18, V-17-11-18, V-19-38-18, V-20-8-18, V-3-19-16, V-4-9-17, V-8-57-17, V-9-1-17—*Anastrophyllum bidens*, *Aneura pinguis*, *Bazzania ovistipula*, *Diplophyllum taxifolium*, *Lepidozia omeiensis*, *Metacalypogeia alternifolia*, *Mnioloma fuscum*, *Mylia vietnamica*, *Plagiochila semidecurrens*, *P. trabeculata*, *Plicanthus hirtellus*, *Scapania contorta*, *S. ornithopoides*, *Schistochilopsis setosa*.

*Herbertus dicranus* (Taylor ex Gottsche, Lindenb. et Nees) Trevis.—1410, 2030, 2835, 2884, 2900, 3105—Open, dry to mesic cliffs, tree trunks and branches, rarely moist decaying wood—**3**, **18**, **23**, **26**, **27**, **31**—V-10-13-22, V-18-21-18, V-2-13-17, V-20-2-18, V-4-27-17, V-8-78-17, V-9-3-17—*Bazzania angustistipula*, *Drepanolejeunea ternatensis*, *Frullania motoyana*, *Heteroscyphus tener*, *Lejeunea flava*, *Plagiochila beddomei*, *P. gracilis*, *P. peculiaris*, *P. semidecurrens*, *Pleurozia subinflata*, *Plicanthus birmensis*, *Radula cavifolia*.

*Herbertus ramosus* (Steph.) H.A. Mill.—1520, 1995, 2030, 2957—Open to partly shaded, mostly moist, rarely mesic cliffs, rarely tree branches—**5**, **10**, **26**, **29**—V-3-48-17, V-7-1-17, V-8-11-17, V-9-1-22—*Bazzania praerupta*, *Calypogeia aeruginosa*, *Cephaloziella willisana*, *Cryptolophocolea sikkimensis*, *Leucolejeunea turgida*, *Metacalypogeia alternifolia*, *Plagiochila hyalodermica*, *P. semidecurrens*, *Scapania ciliatospinosa*, *S. ferruginea*; Phan-si-pan, 2500–3000 m a.s.l. [17].

*Heteroscyphus argutus* (Nees) Schiffn.—1325, 1410, 1520, 1557, 1840, 2030, 2060, 2210—Open to partly shaded moist, rarely mesic, cliffs, boulders and fine soil, mostly near streams, partly shaded moist decaying wood—**1**, **3**, **5**, **8**, **13**, **16**, **18**—V-1-33-17, V-10-24-17, V-2-31-17, V-3-26-17, V-4-34-17, V-5-17-17, V-6-28-17, V-8-20-22—*Bazzania ovistipula*, *B. tridens*, *Calypogeia apiculata*, *C. granulata*, *Delavayella serrata*, *Fuscocephaloziopsis catenulata* subsp. *nipponica*, *Heteroscyphus coalitus*, *Jubula javanica*, *Metzgeria lindbergii*, *Nowellia curvifolia*, *Plagiochila trabeculata*, *Radula apiculata*, *Riccardia parvula*, *Schiffneria hyalina*, *Schistochila doriae*.

*Heteroscyphus coalitus* (Hook.) Schiffn.—1325, 1410, 1520, 1557, 1840, 2014, 2030, 2210, 2670, 2727, 2835—Wet to moist, rarely mesic, cliffs and boulders in part shade to open places, mostly near streams—**1**, **3**, **5**, **8**, **11**, **16**, **18**, **19**, **22**, **31**—V-1-44-17, V-10-13-17, V-10-31-22, V-15-6-18, V-19-32-18, V-2-11-17, V-21-18-18, V-3-17-17, V-4-20-17, V-5-17-17, V-8-27-22—*Bazzania japonica*, *B. ovistipula*, *B. tridens*, *Calypogeia apiculata*, *C. granulata*, *C. lunata*, *Delavayella serrata*, *Fuscocephaloziopsis gollanii*, *Heteroscyphus argutus*, *H. zollingeri*, *Jackiella javanica*, *Lepidozia faurieana*, *L. vitrea*, *Lobatiriccardia yunnanensis*, *Metacalypogeia alternifolia*, *Nowellia curvifolia*, *Pellia endiviifolia*, *Plectocolea tetragona*, *Riccardia decrescens*, *R. flavovirens*, *R. parvula*, *Saccogynidium muricellum*, *Scapania maxima*, *S. ornithopoides*, *Schistochila doriae*, *Trichocolea pluma*, *T. tomentella*; as *Heteroscyphus communis* (Steph.) Schiffn.: Phan-si-pan, 1800, 2400 m a.s.l. [15].

*Heteroscyphus tener* (Steph.) Schiffn.—1840, 1900, 2030, 2060, 2100, 2210, 2610, 2670, 2821, 2835, 2846, 2884, 2900, 2957—Mostly partly shaded to open moist to mesic tree trunks and branches, rarely moist decaying wood and moist cliffs—**8**, **12**, **13**, **16**, **17**, **18**, **20**, **22**, **23**, **24**, **29**, **31**—V-1-50-16, V-2-133-16, V-10-10-22, V-12-18-17, V-16-10-18, V-17-8-18, V-19-18-18, V-20-2-18, V-4-50-17, V-5-39-17, V-6-2-17, V-9-2-17, V-9-6-22—*Bazzania angustistipula*, *B. ovistipula*, *B. pearsonii*, *B. praerupta*, *B. tridens*, *Chiastocaulon fimbriatum*, *Delavayella serrata*, *Drepanolejeunea angustifolia*, *Herbertus dicranus*, *Leucolejeunea turgida*, *Lopholejeunea subfusca*, *Metzgeria leptoneura*, *Plagiochila gracilis*, *P. semidecurrens*, *Plicanthus birmensis*, *P. hirtellus*, *Porella campylophylla*, *Radula inouei*, *R. javanica*, *R. madagascariensis*, *Syzygiella elongella* [4].

* *Heteroscyphus wettsteinii* Schiffner—1565—Mesic limestone cliff in part shade—**15**—V-11-23-22—Comment: the species is somewhat similar from the dorsal view to *Plagiochila nepalensis*, although immediately different in having large and dentate underleaves. The species is characterized by opposite connate dorsally leaves, with three main teeth in the leaf apex and one to several teeth to the base of the ventral side (in conjunction with underleaf). Some features observed in studied specimen are atypical: teeth are present on ventral side of the leaf, underleaf rather shortly bilobed and each lobe is toothed (not simply acute). The “true” *H. wettsteini*i is distributed in Java, Borneo and Papua New Guinea; therefore, the present specimen may belong to another taxon.

*Heteroscyphus zollingeri* (Gottsche) Schiffn.—1520—Open to partly shaded, moist to mesic cliffs near streams—**5**—V-3-71-17– *Calypogeia apiculata*, *Heteroscyphus coalitus*.

*Isotachis japonica* Steph.—1995, 2014, 2060, 2610, 2821, 2100, 2210—Open to partly shaded, most to wet and submerged cliffs and sandy soil near or in the beds of streams, rarely moist decaying wood—**10**, **11**, **12**, **13**, **16**, **20**, **21**—V-11-18-17, V-16-19-18, V-21-7-18, V-6-11-17, V-7-18-17, V-2-143-16, V-5-13-17, V-6-36-17– *Calypogeia granulata*, *C. lunata*, *Cephalozia conchata*, *C. siamensis*, *Jackiella javanica*, *Lepidozia faurieana*, *Scapania ligulata*—Pha-si-Pan, 2700 m a.s.l.; Sapa, 2000 m a.s.l. [13].

*Jackiella javanica* Schiffn.—1325, 1410, 1520, 1557, 2030, 2060, 2210—Open, rarely partly shaded, moist to wet and mesic clayish soil in roadsides, soil covering rocks and cliffs—**1**, **3**, **5**, **13**, **16**, **18**—V-1-13-17, V-2-24-17, V-3-20-17, V-4-43-17, V-5-13-17, V-6-10-17, V-8-22-22—*Calypogeia granulata*, *Cephalozia albula*, *C. siamensis*, *Heteroscyphus coalitus*, *Isotachis japonica*, *Kurzia borneensis*, *Plectocolea ariadne*, *P. tetragona*, *Solenostoma appressifolium*.

*Jubula javanica* Steph.—1325, 1520, 1840, 2210, 2670, 2835, 2846, 2900, 2957—Open to partly shaded moist to wet cliffs and those covered in humus and fine soil—**1**, **5**, **8**, **16**, **22**, **23**, **24**, **29**, **31**—V-1-42-17, V-10-24-17, V-10-32-22, V-17-54-18, V-19-12-18, V-3-13-17, V-5-15-17, V-9-15-22—*Calypogeia cuspidata*, *Fuscocephaloziopsis gollanii*, *Heteroscyphus argutus*, *Metacalypogeia alternifolia*, *Metzgeria leptoneura*, *Plagiochila sciophila*, *Radula apiculata*, *R. kojana*, *Solenostoma suborbiculatum*.

* *Jubula sikkimensis* Steph.—2670, 2846—Open to partly shaded moist to mesic cliffs and boulders near stream and humus covering rocks—**22**, **24**—V-17-1-18, V-19-27-18—*Calypogeia apiculata*, *Metzgeria leptoneura*. Comment: the species is characterized by pyxidate lobules, narrowed to the mouth, and sparse dentation of leaf lobes. The species has highly questionable status and further study is needed to clarify the situation.

* *Kurzia borneensis* Mizut.—1410, 1520, 1557, 1840, 2210, 2610, 2727—Partly shaded, rarely open, moist to mesic cliffs and boulders in narrow valleys, including sites near streams—**3**, **5**, **8**, **16**, **19**, **21**—V-10-12-17, V-11-5-17, V-15-5-18, V-2-24-17, V-3-23-17, V-5-12-17, V-8-3-22—*Fuscocephaloziopsis gollanii*, *Jackiella javanica*, *Plectocolea granulata*, *Saccogynidium muricellum*, *Scapania undulata*.

*Kurzia geniculata* Mizut.—1900—Partly shaded moist humus on steep slope—**8—**V-1-19-16 [4].

*Kurzia lineariloba* Mizut.—2100—Partly shaded moist cliffs—**12**—V-2-111-16 [4].

*Kurzia makinoana* (Steph.) Grolle—1900, 1995, 2014, 2030, 2100, 2210, 2610, 2835, 2900, 3105, 3143—Open to partly shaded cliffs and boulders, including those near streams—**8**, **10**, **11**, **12**, **16**, **17**, **23**, **26**, **27**, **30**, **31**—V-1-77-16, V-2-103-16, V-3-8-16, V-10-17-22, V-12-14-17, V-18-5-18, V-21-17-18, V-5-75-17, V-7-2-17, V-8-31-17, V-9-36-17—*Bazzania angustistipula*, *B. ovistipula*, *Fuscocephaloziopsis gollanii*, *Plagiochila trabeculata*, *Scapania maxima*, *S. metahimalayana*, *S. ornithopoides*, *Schistochilopsis setosa*, *Syzygiella autumnalis* [4].

*Lejeunea alata* Gottsche—2210—Moist decaying branch of fallen tree—**16—**V-5-48-17.

*Lejeunea cocoes* Mitt.—1900—Partly shaded mesic decaying wood—**8**—V-1-32-16 [4].

*Lejeunea eifrigii* Mizut.—2100—Partly shaded thin mesic tree branch—**12**—V-2-15-16 [4].

*Lejeunea flava* (Sw.) Nees—1410, 1520, 1565, 1840, 2030, 2060, 2210—Open to partly shaded mesic, rarely moist, tree trunks and branches of living and decaying trees, including fallen trees—**3**, **5**, **8**, **13**, **15**, **16**, **18**—V-10-47-17, V-11-12-22, V-2-13-17, V-3-55-17, V-4-49-17, V-5-25-17, V-6-38-17—*Acrolejeunea sandvicensis*, *Bazzania angustistipula*, *Drepanolejeunea angustifolia*, *D. ternatensis*, *Frullania obscura*, *F. pocsantha*, *Herbertus dicranus*, *Lejeunea parva*, *L. tuberculosa*, *Leucolejeunea xanthocarpa*, *Lopholejeunea nigricans*, *L. soae*, *L. subfusca*, *Microlejeunea punctiformis*, *Plagiochila beddomei*, *P. gracilis*, *P. pulcherrima*, *Ptychanthus striatus*, *Radula cavifolia*, *R. javanica*, *Spruceanthus semirepandus*.

* *Lejeunea japonica* Mitt.—1520, 2210—Open moist cliffs near streams and partly shaded upper surface of evergreen shrub leaf—**5**, **16—**V-3-69-17, V-5-45-17.

*Lejeunea magohukui* Mizut.—1325, 1620—Partly shaded upper surface of leaf and mesic limestone cliff—**1**, **6—**V-1-6-17, V-13-24-17.

*Lejeunea neelgherriana* Gottsche—1520, 1565, 2030—Mesic partly shaded limestone cliffs, decaying wood, rarely wet boulders near stream—**5**, **15**, **18**—V-11-24-22, V-3-62-17, V-4-28-17—*Drepanolejeunea angustifolia*, *Lejeunea pallidevirens*, *Microlejeunea punctiformis*, *Plagiochila beddomei*, *P. semidecurrens*, *Plicanthus birmensis*, *Porella densifolia*, *P. perrottetiana*, *Radula apiculata*, *R. cavifolia* [8].

*Lejeunea obscura* Mitt.—1325, 1410, 1520, 1565—Open to partly shaded mesic to moist cliffs, branches of living and decaying trees and shrubs, clayish road cuts—**1**, **3**, **5**, **15**—V-1-15-17, V-11-18-22, V-2-20-17, V-3-34-17—*Plagiochila sciophila*.

*Lejeunea pallidevirens* S. Hatt.—1520, 1900, 2030, 2100—Open mesic to moist cliffs near streams, bark of living tree trunks, decaying tree trunk, rarely leaf surfaces—**5**, **8**, **12**, **18**—V-1-23-16, V-2-59-16, V-3-66-17, V-4-57-17—*Lejeunea neelgherriana* [4].

*Lejeunea papilionacea* Steph. Ex Prantl—1325, 1520, 1620—Open mesic tree trunks, roots and limestone cliffs—**1**, **5**, **6**—V-1-1-17, V-13-3-17, V-3-30-17—*Lejeunea tuberculosa*.

*Lejeunea parva* (S. Hatt.) Mizut.—1620, 2030, 2210, 2835, 3050, 3143—Partly shaded moist to mesic decaying wood, branches of living trees, rarely limestone outcrops—**6**, **16**, **18**, **26**, **30**, **31**—V-3-42a-16, V-10-11-22, V-13-29-17, V-4-11-17, V-5-25-17, V-8-1-17—*Lejeunea flava*, *Metzgeria leptoneura*, *Plagiochila semidecurrens*, *P. subtropica*, *Radula apiculata*, *Spruceanthus semirepandus* [4].

*Lejeunea subacuta* Mitt.—1840, 2060—Mesic partly shaded cliffs and open tree trunks—**8**, **13—**V-10-2-17, V-6-30-17.

*Lejeunea tuberculosa* Steph.—1325, 1520, 1840, 2030—Open to partly shaded mesic cliffs and tree trunks—**1**, **5**, **8**, **18**—V-1-1-17, V-10-49-17, V-3-1-17, V-4-23-17—*Drepanolejeunea ternatensis*, *Lejeunea flava*, *L. papilionacea*, *Lopholejeunea eulopha*, *L. nigricans*, *Microlejeunea punctiformis*, *Plagiochila pulcherrima*, *Ptychanthus striatus*.

*Lepidozia faurieana* Steph.—1410, 1520, 1557, 1840, 1900, 1995, 2014, 2030, 2100, 2210, 2670—Open to partly shaded moist to wet cliffs and boulders, including those covered in sandy soil, mostly near watercourses—**3**, **5**, **8**, **10**, **11**, **12**, **16**, **18**, **22**, **26**—V-1-120-16, V-2-118-16, V-10-18-17, V-19-28-18, V-2-19-17, V-21-9-18, V-3-94-17, V-4-15-17, V-5-72-17, V-7-23-17, V-8-17-22—*Bazzania japonica*, *B. tridens*, *Calypogeia aeruginosa*, *C. granulata*, *C. lunata*, *Cephalozia siamensis*, *Heteroscyphus coalitus*, *Isotachis japonica*, *Plagiochila semidecurrens*, *Scapania contorta*, *S. maxima* [4].

*Lepidozia omeiensis* P.C. Chen ex Mizut. et G.C. Zhang—1900, 2727, 2835, 2846, 3105, 3143—Open to partly shaded moist to wet cliffs and boulders, mostly near watercourses—**8**, **5**, **19**, **24**, **27**, **30**, **31—**V-1-125-16, V-3-66-16, V-10-34-22, V-15-30-18, V-17-31-18, V-18-14-18—*Bazzania ovistipula*, *Calypogeia lunata*, *Cephalozia conchata*, *Delavayella serrata*, *Herbertus armitanus*, *Metacalypogeia alternifolia*, *Mnioloma fuscum*, *Mylia vietnamica*, *Plagiochila hyalodermica*, *P. trabeculata*, *Riccardia pumila*, *Schistochilopsis setosa* [4].

*Lepidozia subintegra* Lindenb.—1520—Mesic branch of tree, in part shade—**5**—V-3-57-17 [8].

*Lepidozia subtransversa* Steph.—1995, 2030, 2900, 2957, 3143—Open to partly shaded moist cliffs—**10**, **23**, **26**, **29**, **30**—V-3-90-16, V-7-6-17, V-8-68-17, V-9-17-22, V-9-21-22—*Bazzania angustistipula*, *B. japonica*, *Calypogeia granulata*, *Metacalypogeia alternifolia*, *Plagiochila trabeculata*, *Scapania ciliatospinosa*, *S. ornithopoides*, *Schistochilopsis setosa* [4].

*Lepidozia trichodes* (Reinw., Blume et Nees) Nees—Phan-si-pan, 2100 m a.s.l. [15].

*Lepidozia vitrea* Steph.—1410, 1840, 1900, 2210, 3143—Open to partly shaded cliffs and boulders, including those near streams—**3**, **8**, **16**, **30**—V-1-99-16, V-3-79-16, V-10-29-17, V-2-30-17, V-5-16-17—*Calypogeia apiculata*, *Heteroscyphus coalitus*, *Plectocolea rosulans*, *P. tetragona*, *Riccardia nagasakiensis*, *R. parvula* [4].

*Leptolejeunea balansae* Steph.—2030—Open mesic tree trunk—**18—**V-4-46-17.

*Leptolejeunea elliptica* (Lehm. et Lindenb.) Besch.—1565—Partly shaded mesic leaf of shrub—**15**—V-11-1-22—*Lejeunea flava*.

*Leptolejeunea latifolia* Herzog—2100—Partly shaded thin tree branch—V-2-18k-16—**12** [4].

*Leucolejeunea paroica* N. Kitag.—1900, 2100—Partly shaded tree trunks and branches—**8**, **12**—V-1-56-16, V-2-6-16; as *Cheilolejeunea kitagawae* W. Ye et R.L. Zhu [4].

*Leucolejeunea turgida* (Mitt.) Verd.—1840, 2210, 2957—Open to partly shaded, moist to mesic tree trunk branches, decaying wood and cliffs—**8**, **16**, **29—**V-10-27-17, V-5-51-17, V-9-1-22—*Bazzania angustistipula*, *B. himalayana*, *Chiastocaulon fimbriatum*, *Drepanolejeunea angustifolia*, *Herbertus ramosus*, *Heteroscyphus tener*, *Plagiochila semidecurrens*, *Plicanthus birmensis*, *Radula cavifolia*.

*Leucolejeunea xanthocarpa* (Lehm. et Lindenb.) A. Evans—1620, 2060—Partly shaded mesic tree trunks—**6**, **13**—V-13-19-17, V-6-38-17—*Lejeunea flava*, *Lopholejeunea subfusca*, *Microlejeunea punctiformis*.

*Lobatiriccardia yunnanensis* Furuki et D.G. Long—1520, 1900—Partly shaded wet cliffs near streams, including waterfalls—**5**, **8**—V-1-135-16, V-3-51-17—*Heteroscyphus coalitus*, *Plectocolea tetragona*, *Riccardia decrescens* [4].

*Lophocolea bidentata* (L.) Dumort.—2846—Open moist humus covering cliff—**24—**V-17-52-18.

*Lophocolea heterophylla* (Schrad.) Dumort.—1520—Open moist cliff near stream—**5—**V-3-77-17.

*Lophocolea minor* Nees—2030, 2210—Partly shaded moist to mesic decaying wood—**16**, **18—**V-4-12-17, V-5-22-17.

*Lopholejeunea eulopha* (Taylor) Schiffn.—2030—Partly shaded mesic tree trunk—**18**—V-4-66-17—*Lejeunea tuberculosa*.

*Lopholejeunea nigricans* (Lindenb.) Steph. ex Schiffn.—1840, 2030—Open to partly shaded mesic tree trunks—**8**, **18**—V-10-49-17, V-4-68-17—*Lejeunea flava*, *L. tuberculosa*, *Microlejeunea punctiformis*, *Plagiochila nepalensis*, *P. pulcherrima*, *Porella acutifolia*, *Ptychanthus striatus*; Sapa, 1600, 1700 m a.s.l. [17].

*Lopholejeunea soae* R.L. Zhu et Gradst.—1840—Open mesic tree trunks—**8**—V-10-43-17—Chiastocaulon fimbriatum, Drepanolejeunea angustifolia, Lejeunea flava, Microlejeunea punctiformis, Radula cavifolia.

*Lopholejeunea subfusca* (Nees) Schiffn.—1325, 1565, 2060—Partly shaded mesic tree trunks, rarely moist decaying wood—**1**, **13**, **15**—V-1-12-17, V-11-9-22, V-6-32-17—*Chiastocaulon fimbriatum*, *Drepanolejeunea angustifolia*, *Heteroscyphus tener*, *Lejeunea flava*, *Leucolejeunea xanthocarpa*, *Microlejeunea punctiformis*, *Plagiochila semidecurrens*, *Plicanthus birmensis*, *Syzygiella elongella*.

*Lopholejeunea zollingeri* (Prantl) Steph. ex Schiffn.—2100—Partly shaded tree trunk—**12**—V-2-50-16 [4].

*Makinoa crispata* (Steph.) Miyake—1557—Open moist sandy soil and wet cliffs near streams—**5**—V-8-14-22.

*Marchantia emarginata* Reinw., Blume et Nees ssp. *tosana* (Steph.) Bischl.—1325, 1520, 1620—Open mesic to moist sandy soil on road cuts and steep slopes, partly shaded cliff crevices in limestone outcrops—**1**, **5**, **6—**V-1-14-17, V-13-7-17, V-3-12-17.

*Marchantia paleacea* Bertol.—1325, 1350, 1800—Open mesic to moist road cuts and rice field edges—**1**, **2**, **4—**V-1-20-17, V-10a-2-17, V-4-15-16.

*Marchantia papillata* subsp. *grossibarba* (Steph.) Bischl.—1350, 1557, 1900—Open moist to wet cliffs (including those of limestone) and boulders near stream—**2**, **5**, **8**—V-1-4-16, V-4-3-16, V-8-42-22 [4].

*Marchantia polymorpha* L.—3105—Open moist humus on slope—**27—**V-18-8-18.

*Marsupella stoloniformis* N. Kitag.—2610, 2727, 2821, 2835, 2957—Open, rarely partly shaded, mesic, moist and wet cliffs near streams—**19**, **20**, **21**, **29**, **31**—V-10-28-22, V-11-11-17, V-15-22-18, V-16-17-18, V-9-44-22—*Marsupella vietnamica*, *Scapania ligulata*, *Solenostoma suborbiculatum*, *Sphenolobopsis pearsonii*.

*Marsupella vietnamica* Bakalin et Fedosov—1840, 1995, 2610, 2727, 2821, 2900—Open, rarely partly shaded moist to wet cliffs and boulders near streams and in the streambeds—**8**, **10**, **12**, **19**, **20**, **21**, **23**, **31**—V-10-22-17, V-11-15a-17, V-15-34-18, V-16-22-18, V-7-13-17, V-9-33-17, V-10-27-22, V-2-101-16—*Bazzania ovistipula*, *Cephalozia conchata*, *Chiastocaulon theriotianum*, *Cylindrocolea recurvifolia*, *Marsupella stoloniformis*, *Scapania contorta*, *S. undulata*, *Solenostoma suborbiculatum*, *S. faurieanum*.

*Mastigophora diclados* (Brid. ex F. Weber) Nees—1520—Partly shaded mesic tree branch—**5—**V-3-54-17.

*Mastigophora woodsii* (Hook.) Nees—1995, 2014—Open moist cliffs near stream—**10**, **11**—V-21-21-18, V-7-12-17 [8].

*Metacalypogeia alternifolia* (Nees) Grolle—1840, 1995, 2030, 2210, 2610, 2670, 2821, 2846, 2900, 2957, 3105, 3143—Open to partly shaded mesic to moist cliffs and boulders, including those near streams—**8**, **10**, **16**, **17**, **20**, **21**, **22**, **23**, **24**, **26**, **27**, **29**, **30**—V-10-1-17, V-11-1-17, V-12-9-17, V-16-13-18, V-17-23-18, V-18-15-18, V-19-29-18, V-3-19-16, V-5-19-17, V-7-7-17, V-8-20-17, V-9-11-22, V-9-74-22—*Anastrophyllum bidens*, *Bazzania angustistipula*, *B. japonica*, *B. ovistipula*, *B. praerupta*, *Calycularia crispula*, *Calypogeia aeruginosa*, *C. granulata*, *Cryptolophocolea sikkimensis*, *Delavayella serrata*, *Fuscocephaloziopsis gollanii*, *Herbertus armitanus*, *H. ramosus*, *Heteroscyphus coalitus*, *Jubula javanica*, *Lepidozia omeiensis*, *L. subtransversa*, *Metzgeria leptoneura*, *M. lindbergii*, *Mnioloma fuscum*, *Mylia vietnamica*, *Plagiochila hyalodermica*, *P. sciophila*, *P. semidecurrens*, *P. trabeculata*, *Plicanthus hirtellus*, *Pseudolepicolea andoi*, *Scapania ciliatospinosa*, *S. ferruginea*, *S. ligulata*, *S. maxima*, *S. metahimalayana*, *S. ornithopoides*, *Schistochilopsis setosa*, *Solenostoma faurieanum* [4].

*Metzgeria consanguinea* Schiffn.—1410, 2100, 3143—Mesic open cliffs and partly shaded thin branches of trees—**3**, **12**, **28**, **30—**V-2-17a-16, V-2-8-17, V-3-42d-16.

*Metzgeria leptoneura* Spruce—2670, 2821, 2835, 2846, 2900, 2957—Open to partly shaded mesic to moist cliffs and boulders, including those covered in humus and near streams, rarer mesic tree trunks in part shade—**20**, **22**, **23**, **24**, **29**, **31**—V-10-11-22, V-16-25-18, V-17-17-18, V-19-44-18, V-9-13-22, V-9-19-17—*Bazzania angustistipula*, *Fuscocephaloziopsis gollanii*, *Heteroscyphus tener*, *Jubula javanica*, *J. sikkimensis*, *Lejeunea parva*, *Metacalypogeia alternifolia*, *Plagiochila sciophila*, *P. semidecurrens*, *Porella campylophylla* [8].

*Metzgeria lindbergii* Schiffn.—1520, 1840, 2210—Open to shaded moist cliffs in deep valleys, including those near streams—**5**, **8**, **16**—V-10-1-17, V-3-33-17, V-5-65-17—*Heteroscyphus argutus*, *Metacalypogeia alternifolia*, *Plagiochila flexuosa*, *P. trabeculata*, *Porella campylophylla*; as *Metzgeria conjugata* subsp. *japonica* (S. Hatt.) Kuwah.: Phan-si-pan, 2400 m a.s.l. [15].

*Metzgeria pubescens* (Schrank) Raddi—as *Apometzgeria pubescens* (Schrank) Kuwah.: Phan-si-pan, 2400 m a.s.l. [15].

*Microlejeunea punctiformis* (Taylor) Steph.—1840, 2030, 2060—Open to partly shaded mesic tree trunks and decaying wood—**8**, **13**, **18**—V-10-47-17, V-4-28-17, V-6-38-17—*Drepanolejeunea angustifolia*, *Lejeunea flava*, *L. neelgherriana*, *L. tuberculosa*, *Leucolejeunea xanthocarpa*, *Lopholejeunea nigricans*, *L. soae*, *L. subfusca*, *Plagiochila beddomei*, *P. semidecurrens*, *Plicanthus birmensis*, *Ptychanthus striatus*, *Radula* cavifolia; Sapa 1650, Phan-si-pan 2400 [15].

*Mnioloma fuscum* (Lehm.) R.M. Schust.—1557, 2100, 2610, 2846—Open mesic to moist cliffs and boulders, including those near streams—**5**, **12**, **21**, **24**—V-2-106-16, V-11-4-17, V-17-31-18, V-8-13-22—*Aneura pinguis*, *Bazzania praerupta*, *Herbertus armitanus*, *Lepidozia omeiensis*, *Metacalypogeia alternifolia*, *Plagiochila semidecurrens*, *Scapania ornithopoides*, *Schistochilopsis setosa* [4].

*Mylia vietnamica* Bakalin et Vilnet—2846, 2884, 2900, 3105, 3143—Open to partly shaded moist to mesic cliffs—**23**, **24**, **27**, **30**—V-17-11-18, V-18-14-18, V-20-8-18, V-3-17a-16, V-9-16-17—*Anastrophyllum bidens*, *Aneura pinguis*, *Bazzania ovistipula*, *Herbertus armitanus*, *Lepidozia omeiensis*, *Metacalypogeia alternifolia*, *Plicanthus hirtellus*, *Scapania contorta*, *S. ornithopoides* [12].

* *Neolepidozia papulosa* (Steph.) Fulford et J.Taylor—1557—Partly shaded mesic boulder—**5—**V-8-5-22.

*Neolepidozia wallichiana* (Gottsche) Fulford et J. Taylor—2100—Partly shaded mesic decaying wood—**12**—V-2-76-16 [4].

*Notoscyphus lutescens* (Lehm. et Lindenb.) Mitt.—1520—Open to partly shaded mesic sandstone cliffs near watercourses—**5—**V-3-27-17.

*Nowellia curvifolia* (Dicks.) Mitt.—1900, 2030, 2060, 2100, 2210, 2610—Partly shaded moist decaying wood, open mesic cliffs, rarely open mesic tree trunks and partly shaded tree roots—**8**, **12**, **13**, **16**, **17**, **18**—V-1-29-16, V-2-70-16, V-12-21-17, V-4-30-17, V-5-21-17, V-6-16-17—*Delavayella serrata*, *Heteroscyphus argutus*, *H. coalitus* [4].

*Odontoschisma grosseverrucosum* Steph.—1557—Partly shaded mesic boulder—**5—**V-8-4-22.

*Pallavicinia levieri* Schiffn.—1520, 2821—Open to partly shaded moist to wet boulders along stream—**5**, **20—**V-16-15-18, V-3-60-17.

*Pallavicinia subciliata* (Austin) Steph.—1325, 1350, 1410, 1557, 1840, 1900, 2060, 2100—Open to partly shaded moist to wet cliffs and boulders also covered in soil, sandy and fine soils mostly along streams—**1**, **2**, **3**, **5**, **8**, **12**, **13**—V-1-11-16, V-1-32-17, V-10-9-17, V-2-16-17, V-2-2-16, V-4-10-16, V-6-5-17, V-8-25-22 [4].

*Pellia endiviifolia* (Dicks.) Dumort.—1325, 1520, 1557, 1840—Open to partly shaded moist to wet cliffs and soil, mostly along streams—**1**, **5**, **8**—V-1-38-17, V-10-30-17, V-3-3-17, V-8-33-22—*Dumortiera hirsuta*, *Heteroscyphus coalitus*.

*Plagiochasma cordatum* Lehm. et Lindenb.—1350, 1565—Open to partly shaded mesic to dry limestone cliffs and their detritus—**2**, **15**—V-11-2-22, V-4-36-16 [4].

*Plagiochasma japonicum* (Steph.) C. Massal.—1350, 1620—Open dry to mesic limestone cliffs and limestone detritus—**2**, **6**—V-13-33-17, V-4-26-16 [4].

*Plagiochasma rupestre* (J.R. Forst. et G. Forst.) Steph.—1350—Partly shaded mesic limestone cliff crevices—**2—**V-4-7-16.

*Plagiochila assamica* Steph.—1900, 2030, 2100—Partly shaded mesic tree trunk—**8**, **12**, **18**—V-1-48-16, V-2-45-16, V-4-60-17—*Bazzania japonica*, *B. ovistipula*, *B. tridens* [4].

*Plagiochila beddomei* Steph.—1565, 1840, 2030, 2210—Open to partly shaded mesic cliffs (including those of limestone), decaying wood and rarely tree trunk—**8**, **15**, **16**, **18**, **26**—V-10-4-17, V-11-29-22, V-4-28-17, V-5-40-17, V-8-27-17—*Bazzania angustistipula*, *B. ovistipula*, *Drepanolejeunea angustifolia*, *Frullania moniliata*, *Herbertus dicranus*, *Lejeunea flava*, *L. neelgherriana*, *Microlejeunea punctiformis*, *Plagiochila gracilis*, *P. semidecurrens*, *Plicanthus birmensis*, *P. hirtellus*, *Radula cavifolia*, *Scapania maxima* [8].

*Plagiochila chenii* Grolle et M.L. So—1900—Partly shaded mesic tree trunk—**8—**V-1-62-16 [4].

*Plagiochila chinensis* Steph.—as *Plagiochila wilsoniana* Steph.: Sapa, 1785 m a.s.l. [17].

*Plagiochila devexa* Steph.—2100—Partly shaded mesic cliffs—**12**—V-2-117a-16 [4].

*Plagiochila durelii* Schiffn.—2030, 2610, 2670, 2835—Open to partly shaded moist to mesic cliffs, tree trunks, branches and decaying wood—**17**, **18**, **22**, **26**, **31—**V-10-4-22, V-12-1-17, V-19-14-18, V-4-69-17, V-8-8-17.

*Plagiochila flexuosa* Mitt.—1325, 1520, 1557, 1620, 2014, 2210—Open to partly shaded moist to wet, rarely mesic, cliffs—**1**, **5**, **11**, **16—**V-1-26-17, V-21-6-18, V-3-93-17, V-5-61-17, V-8-28-22—*Metzgeria lindbergii*, *Porella campylophylla*; Phan-si-pan, 2400 m a.s.l. [15].

*Plagiochila fruticosa* Mitt.—1840, 2030, 2210, 2670—Open to partly shaded cliffs and boulders, including those near stream, mesic tree trunks—**8**, **16**, **18**, **22**—V-10-10-17, V-19-45-18, V-4-37-17, V-5-35-17—*Cololejeunea haskarliana*, *Plagiochila pulcherrima*, *Plectocolea rosulans*; Phan-si-pan, 2400 m a.s.l. [15]; Sapa, 1785, 2000 m a.s.l. [17].

*Plagiochila gracilis* Lindenb. et Gottsche—1840, 2030, 2100, 2727, 2821, 2957, 3143—Open to partly shaded mesic tree trunks (including bamboo stems) and branches, rarely decaying wood and moist cliffs—**8**, **12**, **18**, **19**, **20**, **29**, **30**—V-2-29-16, V-3-50-16, V-10-50-17, V-15-14-18, V-16-3-18, V-4-49-17, V-9-3-22—*Bazzania angustistipula*, *B. ovistipula*, *Herbertus dicranus*, *Heteroscyphus tener*, *Lejeunea flava*, *Plagiochila beddomei*, *P. semidecurrens*, *Radula cavifolia*, *Schistochilopsis setosa* [4].

*Plagiochila grollei* Inoue—Phan-si-pan, 2560 m a.s.l. [17].

* *Plagiochila hokinensis* Steph.—1520—Open moist boulder near stream—**5**—V-3-9-17. Comment: the name is regarded as a synonym for *P. chinensis* Steph. by So [28]. However, the type of *P. chinenesis* (G20043/00064139!) has more orbicular leaves than that drawn by So [28] and rather comparable with *P. ovalifolia* (if not another taxon). The treatment of *P. chinensis* by So [28] coincides with the plant in the type of *P. hokinensis*, as we found after a study of the type specimen (G35918/00061465!). Currently, we believe that these are two different taxa and the synonymizing of the names should be revised.

* *Plagiochila hyalodermica* Grolle et M. L. So—2030, 2670, 2957, 3105—Open to partly shaded moist cliffs—**22**, **26**, **27**, **29**—V-18-17-18, V-19-20-18, V-8-15-17, V-9-19-22—*Bazzania praerupta*, *B. tridens*, *Calycularia crispula*, *Calypogeia aeruginosa*, *C. granulata*, *C. sinensis*, *Cephaloziella willisana*, *Cryptolophocolea sikkimensis*, *Delavayella serrata*, *Fuscocephaloziopsis gollanii*, *Herbertus ramosus*, *Lepidozia omeiensis*, *Metacalypogeia alternifolia*, *Plagiochila sciophila*, *P. semidecurrens*, *Plicanthus birmensis*, *Scapania ciliatospinosa*, *S. ferruginea*, *Schistochilopsis setosa*.

*Plagiochila junghuhniana* Sande Lac.—1565—Partly shaded mesic limestone cliffs—**15**—V-11-21-22– *Radula apiculata*. Comment: *Plagiochila junghuhniana* is characterized by oblong leaves, widest above base (ca. 1/3), thin walls with large trigones, unclearly incised leaf apex. This is the tropical element in the flora and there are some doubts that this species is in hand in North Vietnam.

*Plagiochila khasiana* Mitt.—2210—Partly shaded mesic tree trunk—**16—**V-5-79-17.

*Plagiochila nepalensis* Lindenb.—1565, 2030—Open to partly shaded mesic limestone cliffs and tree trunks—**15**, **18**—V-11-25-22, V-4-45-17; as *Plagiochila gollanii* Steph.: Sapa, 1650 m a.s.l. [17].

*Plagiochila oblonga* Inoue—Phan-si-pan, 2000–3000 m a.s.l. [17]; as *Plagiochila* cf. *oblonga*: Phan-si-pan [15].

*Plagiochila ovalifolia* Mitt.—1900, 2210—Partly shaded moist to wet cliffs, also near waterfalls—**8**, **16**—V-1-136-16, V-5-64-17 [4].

*Plagiochila parvifolia* Lindenb.—1325—Partly shaded mesic tree branch—**1**—V-1-4-17—*Ptychanthus striatus*; as *Plagiochila* cf. *ventricosa* Steph.: Sapa, 1600 m a.s.l. [17].

*Plagiochila peculiaris* Schiffn.—1410, 1520—Open to partly shaded mesic tree branches and decaying wood—**3**, **5**—V-2-12-17, V-3-38-17—*Cheilolejeunea imbricata*, *Herbertus dicranus*, *Porella caespitans*, *Ptychanthus striatus*; as *Plagiochila crassitexta* Steph. (probably means that it is var. *nakaiana* (S. Hatt.) S. Hatt.): Sapa, 1700 m a.s.l. [17].

*Plagiochila pseudofirma* Herzog—1900, 2030, 3143—Open to partly shaded moist cliffs, decaying wood, tree trunks—**12**, **18**, **26**, **30**—V-2-42-16, V-3-107-16, V-4-48-17, V-8-10-17—*Bazzania angustistipula*, *Scapania ciliatospinosa*, *S. ornithopoides* [4].

*Plagiochila pulcherrima* Horik.—1520, 2030, 2060, 2210, 2670—Open to partly shaded mesic tree trunks, decaying wood, open dry cliffs and moist sandy stream bank—**5**, **13**, **16**, **18**, **22**—V-19-23-18, V-3-39-17, V-4-53-17, V-5-42-17, V-6-39-17—*Drepanolejeunea ternatensis*, *Frullania nepalensis*, *Lejeunea flava*, *L. tuberculosa*, *Lopholejeunea nigricans*, *Plagiochila fruticosa*, *Porella acutifolia*, *P. japonica*.

*Plagiochila salacensis* Gottsche—1620—Partly shaded mesic limestone cliffs—**6—**V-13-23-17.

*Plagiochila sciophila* Nees ex Lindenb.—1325, 1565, 2210, 2670, 2835, 2957—Open to partly shaded mesic to moist tree trunks, decaying wood, cliffs, humus covering rocks—**1**, **15**, **16**, **22**, **29**, **31**—V-1-18-17, V-10-9-22, V-11-37-22, V-19-48-18, V-5-23-17, V-9-11-22—*Bazzania angustistipula*, *Calypogeia granulata*, *Fuscocephaloziopsis gollanii*, *Jubula javanica*, *Lejeunea obscura*, *Metacalypogeia alternifolia*, *Metzgeria leptoneura*, *Plagiochila hyalodermica*, *Ptychanthus striatus*, *Radula apiculata*, *Riccardia parvula*—Sapa, 1600, 1700–1800 m a.s.l. [17].

*Plagiochila secretifolia* Mitt.—Hoang-lien-son, Mt. Phan-si-pan [28].

*Plagiochila semidecurrens* (Lehm. et Lindenb.) Lehm. et Lindenb.—1995, 2030, 2060, 2210, 2610, 2670, 2727, 2835, 2884, 2900, 2957, 3050, 3105—Open to partly shaded mesic to moist tree trunks, decaying wood, cliffs, including those near streams—**10**, **13**, **16**, **18**, **17**, **18**, **19**, **21**, **22**, **23**, **26**, **27**, **29**, **31**—V-10-10-22, V-11-3-17, V-12-15-17, V-15-14-18, V-18-21-18, V-19-14-18, V-20-11-18, V-4-24-17, V-5-1-17, V-6-32-17, V-7-8-17, V-8-11-17, V-9-11-17, V-9-58-22—*Acrobolbus ciliatus*, *Aneura pinguis*, *Bazzania himalayana*, *B. ovistipula*, *B. pearsonii*, *B. praerupta*, *B. tridens*, *Calypogeia vietnamica*, *Chiastocaulon fimbriatum*, *Cryptolophocolea sikkimensis*, *Delavayella serrata*, *Drepanolejeunea angustifolia*, *Frullania nepalensis*, *F. yuennanensis*, *Herbertus armitanus*, *H. dicranus*, *H. ramosus*, *Heteroscyphus tener*, *Lejeunea neelgherriana*, *L. parva*, *Lepidozia faurieana*, *Leucolejeunea turgida*, *Lopholejeunea subfusca*, *Metacalypogeia alternifolia*, *Metzgeria leptoneura*, *Microlejeunea punctiformis*, *Mnioloma fuscum*, *Plagiochila beddomei*, *P. durelii*, *P. gracilis*, *P. hyalodermica*, *P. trabeculata*, *Plicanthus birmensis*, *Radula cavifolia*, *R. inouei*, *R. madagascariensis*, *Scapania ciliatospinosa*, *S. ferruginea*, *S. ornithopoides*, *Schistochilopsis setosa*, *Spruceanthus semirepandus*, *Syzygiella elongella*; Phan-si-pan, 2100 m a.s.l. [15]; as *Plagiochila vietnamica* Inoue (the conspecificity may be questioned): in silvis muscosis sub cacumine; “Phan Si San”, 2500–3000 m a.s.l. [29].

*Plagiochila subtropica* Steph.—1565, 1840, 1900, 2030, 2100, 2210—Open to partly shaded mesic tree trunks, branches and decaying wood, rarely mesic cliffs—**8**, **12**, **15**, **16**, **18**—V-1-87-16, V-2-10-16, V-10-46-17, V-11-10-22, V-4-54-17, V-5-50-17—*Acrolejeunea infuscata*, *Frullania yuennanensis*, *Lejeunea parva*, *Ptychanthus striatus* [4].

*Plagiochila teysmannii* Sande Lac.—1900—Open mesic tree trunk—**8**—V-1-36-16 [4].

*Plagiochila trabeculata* Steph.—1520, 2030, 2210, 2670, 2727, 2835, 2846, 2900, 2957—Open to partly shaded moist to mesic cliffs, boulders, decaying wood, rarely tree trunks—**5**, **16**, **18**, **19**, **22**, **23**, **24**, **26**, **29**, **31**—V-10-17-22, V-10-20-22, V-15-45-18, V-17-45-18, V-19-40-18, V-3-33-17, V-4-32-17, V-4-32a-17, V-4-52-17, V-4-67-17, V-5-31-17, V-8-19-17, V-9-20-22, V-9-25-17, V-9-39-22, V-9-4-22, V-9-42-22, V-9-52-22, V-9-52a-22, V-9-52b-22—*Bazzania angustistipula*, *B. ovistipula*, *B. tridens*, *Calycularia crispula*, *Calypogeia cuspidata*, *C. granulata*, *Herbertus armitanus*, *Heteroscyphus argutus*, *Kurzia makinoana*, *Lepidozia omeiensis*, *L. subtransversa*, *Metacalypogeia alternifolia*, *Metzgeria lindbergii*, *Plagiochila semidecurrens*, *Porella campylophylla*, *Scapania metahimalayana*, *Schistochilopsis setosa*; as *Plagiochila pocsii* Inoue: in silvis sub cacumine; Phan-Si-Pan, 2500–3000 m a.s.l. [29].

*Plagiochila vexans* Schiffn. ex Steph.—2100—Partly shaded mesic decaying wood—**12**—V-2-71-16 [4].

* *Plagiochila zhuensis* Grolle et M. L. So—2821—Partly shaded mesic *Sinobambusa* trunk—**20—**V-16-28-18.

*Plectocolea ariadne* (Taylor ex Lehm.) Mitt.—1325—Open moist clayish road cut—**1**—V-1-13-17—*Jackiella javanica*.

* *Plectocolea granulata* (Steph.) Bakalin—1325, 1410, 1557—Open to partly shaded mesic cliffs and boulders—**1**, **3**, **5**—V-1-28-17, V-2-10-17, V-8-3-22—*Bazzania tridens*, *Calypogeia granulata*, *Kurzia borneensis*, *Plectocolea rosulans*, *Riccardia parvula*.

*Plectocolea hasskarliana* (Nees) Mitt.—1557—Open moist clayish trail side—**5**—V-8-21-22—*Plectocolea tetragona*, *P. truncata*.

*Plectocolea infusca* Mitt.—1410—Open moist cliff crevice near stream—**3—**V-2-3-17.

*Plectocolea rosulans* (Steph.) S. Hatt.—1325, 1410, 1520, 1840, 2210—Open to partly shaded moist to wet cliffs, mostly near streams—**1**, **3**, **5**, **8**, **16**—V-1-46-17, V-10-10-17, V-2-10-17, V-3-52-17, V-5-74-17—*Calypogeia granulata*, *Lepidozia vitrea*, *Plagiochila fruticosa*, *Plectocolea granulata*, *Riccardia nagasakiensis*, *R. parvula*, *Scapania maxima*.

*Plectocolea tetragona* (Lindenb.) Amakawa—1410, 1520, 1557—Open, rarely partly shaded, cliffs near streams, rarely clayish trail sides—V-2-11-17, V-3-15-17, V-8-22-22—**3**, **5**—*Calypogeia apiculata*, *Heteroscyphus coalitus*, *Jackiella javanica*, *Lepidozia vitrea*, *Lobatiriccardia yunnanensis*, *Plectocolea hasskarliana*, *P. truncata*, *Riccardia parvula*.

*Plectocolea truncata* (Nees) Bakalin—1410, 1520, 1557—Open to partly shaded moist cliffs, including those near streams, trail sides—**3**, **5**—V-2-17-17, V-3-2-17, V-8-11-22—*Plectocolea hasskarliana*, *P. tetragona*.

*Pleurozia gigantea* (F. Weber) Lindb.—2030, 2060, 2210—Open to partly shaded mesic to moist decaying wood and mesic boulders—**13**, **16**, **18**—V-4-36-17, V-5-36-17, V-6-33-17—*Bazzania praerupta*, *Plicanthus birmensis*; Sapa, 1500, 2000 m a.s.l. [13].

*Pleurozia subinflata* (Austin) Austin—1840, 2030, 2835, 2884, 2900, 3105—Open to partly shaded mesic tree trunks, branches and cliffs—**8**, **18**, **23**, **25**, **27**, **31**—V-10-14-22, V-10-40-17, V-18-1-18, V-20-4-18, V-4-39-17, V-9-12-17—*Bazzania angustistipula*, *Herbertus dicranus*, *Radula cavifolia*.

*Plicanthus birmensis* (Steph.) R.M. Schust.—2030, 2060, 2210, 2670, 2900, 2957, 3105—Open, rarely partly shaded, mesic, rarely moist, tree trunks, decaying wood, cliffs—**13**, **16**, **18**, **22**, **23**, **27**, **29**—V-18-21-18, V-19-7-18, V-4-28-17, V-5-1-17, V-6-14-17, V-9-14-17—*Bazzania pearsonii*, *B. praerupta*, *Chiastocaulon fimbriatum*, *Drepanolejeunea angustifolia*, *Frullania nepalensis*, *Herbertus dicranus*, *Heteroscyphus tener*, *Lejeunea neelgherriana*, *Leucolejeunea turgida*, *Lopholejeunea subfusca*, *Microlejeunea punctiformis*, *Plagiochila beddomei*, *P. hyalodermica*, *P. semidecurrens*, *Pleurozia gigantea*, *Radula cavifolia*, *R. javanica*, *Syzygiella elongella*.

*Plicanthus hirtellus* (F. Weber) R.M. Schust.—1840, 2060, 2210, 3143—Open to partly shaded mesic to moist tree trunks, decaying wood and boulders—**8**, **13**, **16**, **30**—V-10-44-17, V-3-19-16, V-5-3-17, V-6-35-17—*Herbertus armitanus*, *Heteroscyphus tener*, *Metacalypogeia alternifolia*, *Mylia vietnamica*, *Plagiochila beddomei*; as *Chandonanthus hirtellus* (F. Weber) Mitt.: Sapa, 1500 m a.s.l. [17].

*Porella acutifolia* (Lehm. et Lindenb.) Trevis.—1900, 2030, 2100—Open to partly shaded mesic, rarely moist, cliffs and tree trunks—**8**, **12**, **18**—V-1-87b-16, V-2-141-16, V-4-4-17—*Lopholejeunea nigricans*, *Plagiochila pulcherrima*.

*Porella caespitans* (Steph.) S. Hatt.—1350, 1410, 1620—Open to partly shaded mesic to dry limestone cliffs—**2**, **3**, **6—**V-13-26-17, V-2-12-17, V-4-33-16—*Cheilolejeunea imbricata*, *Plagiochila peculiaris*, *Porella perrottetiana*, *Ptychanthus striatus*; as *Porella piligera* Steph. ex Pócs: Sapa, 1650 m a.s.l. [15]; as *Porella urophylla* fo. *setigera* (Steph.) Pócs: Sapa 1500, 1600 m a.s.l. [14].

*Porella campylophylla* (Lehm. et Lindenb.) Trevis.—1520, 1900, 2030, 2210, 2610, 2670, 2835, 2957—Open, rarely partly shaded, mesic to moist cliffs, decaying wood and tree trunks—**5**, **8**, **16**, **17**, **18**, **22**, **29**, **31**—V-1-88-16, V-10-19-22, V-12-22-17, V-19-24-18, V-3-43-17, V-4-52-17, V-5-5-17, V-9-45-22—*Heteroscyphus tener*, *Metzgeria leptoneura*, *M. lindbergii*, *Plagiochila flexuosa*, *P. trabeculata*.

*Porella densifolia* (Steph.) S. Hatt.—1565, 1620—Open to partly shaded mesic limestone cliffs—**6**, **15**—V-11-24-22, V-13-32-17—*Lejeunea neelgherriana*, *Porella perrottetiana*, *Radula apiculata*—as *Porella densifolia* var. *paraphyllina* (P.C. Chen) Pócs: Sapa, 1750 m a.s.l. [14].

*Porella grandifolia* (Steph.) S. Hatt.—Sapa, 1600 m a.s.l. [14].

*Porella japonica* (Sande Lac.) Mitt.—2030—Partly shaded mesic tree trunk—**18**—V-4-72-17—*Plagiochila pulcherrima*; as *Porella japonica*: Sapa, 1725 m a.s.l. [14].

*Porella obtusata* (Taylor) Trevis. f. *macroloba* (Steph.) S. Hatt.—1565, 1620—Open to partly shaded dry to mesic limestone cliffs, rarely tree trunks—**2**, **6**, **15**—V-11-31-22, V-13-25-17, V-4-30-16; as *Porella thuja* (Dicks.) Lindb.: Sapa, 1650–1800 m a.s.l. [14].

*Porella perrottetiana* (Mont.) Trevis.—1565, 1620—Open to partly shaded mesic limestone cliffs—V-11-24-22, V-13-26-17—**6**, **15**—*Lejeunea neelgherriana*, *Porella caespitans*, *P. densifolia*, *Radula apiculata*; as *Porella perrottetiana* var. *angustifolia* Pócs: Sapa 1500, 1600, 1650–1800 m a.s.l.; 2573 m a.s.l. (type of var. *angustifolia*) [14].

*Porella plumosa* (Mitt.) Parihar—Phan-si-pan, 2400 [15]; as *Porella madagascariensis* f. i*ntegristipula* Pócs: Hoang-Lien-Son, 2000 m a.s.l. [14].

*Porella reflexigastria* Pócs—1565—Partly shaded mesic limestone cliff—**15**—V-11-22-22; Sapa, 1650, 1700 m a.s.l. [14]; as *Porella piligera* var. *grossedentata* Pócs: Sapa 1900, 1650, 1600 m a.s.l. [14].

*Preissia quadrata* (Scop.) Nees—1900—Open moist cliff—**8**—V-1-110-16—*Aneura pinguis*.

*Pseudolepicolea andoi* (R.M. Schust.) Inoue—1995, 2210—Open to partly shaded moist cliffs—**10**, **16**—V-5-68-17, V-7-7-17—*Metacalypogeia alternifolia* [8].

*Ptychanthus striatus* (Lehm. et Lindenb.) Nees—1325, 1410, 1520, 1565, 1620, 1840, 2030—Open to partly shaded mesic tree trunks and branches and cliffs, including those of limestone—**1**, **3**, **5**, **6**, **8**, **15**, **18**—V-1-4-17, V-10-14-17, V-11-36-22, V-13-21-17, V-2-12-17, V-3-22-17, V-4-59-17—*Cheilolejeunea imbricata*, *Frullania yuennanensis*, *Lejeunea flava*, *L. tuberculosa*, *Lopholejeunea nigricans*, *Microlejeunea punctiformis*, *Plagiochila parvifolia*, *P. peculiaris*, *P. sciophila*, *P. subtropica*, *Porella caespitans*; Sapa, 1600–1780 m a.s.l. [13].

*Radula acuminata* Steph.—1410—Open leaf of evergreen shrub upper surface—**3**– V-2-6-17.

*Radula apiculata* Sande Lac. ex Steph.—1325, 1350, 1565, 1620, 1840—Partly shaded mesic to moist, rarely wet, cliffs, including those of limestone and near streams—**1**, **2**, **6**, **8**, **15**—V-4-21-16, V-1-42-17, V-10-17-17, V-11-21-22, V-13-27-17—*Heteroscyphus argutus*, *Jubula javanica*, *Lejeunea neelgherriana*, *L. parva*, *Plagiochila junghuhniana*, *P. sciophila*, *Porella densifolia*, *P. perrottetiana* [4].

*Radula assamica* Steph.—Sapa, 1600 m a.s.l. [13].

*Radula cavifolia* Hampe—1410, 1840, 2030, 2727, 2835, 2846, 2900, 2957, 3105—Open to partly shaded mesic, rarely moist cliffs, tree trunks and branches, decaying wood—**3**, **8**, **18**, **19**, **23**, **24**, **27**, **29**, **31**—V-10-13-22, V-10-37-17, V-15-1-18, V-17-2-18, V-18-3-18, V-2-13-17, V-4-28-17, V-9-11-17, V-9-5-22—*Bazzania angustistipula*, *B. ovistipula*, *Drepanolejeunea angustifolia*, *D. ternatensis*, *Frullania tagawana*, *Fuscocephaloziopsis gollanii*, *Herbertus dicranus*, *Lejeunea flava*, *L. neelgherriana*, *Leucolejeunea turgida*, *L. soae*, *Microlejeunea punctiformis*, *Plagiochila beddomei*, *P. gracilis*, *P. semidecurrens*, *Pleurozia subinflata*, *Plicanthus birmensis*, *Scapania ciliatospinosa*, *S. ornithopoides*, *Schiffneria hyalina*, *Sphenolobopsis pearsonii*, *Syzygiella elongella*, *Vietnamiella epiphytica*.

* *Radula constricta* Steph.—1620—Open dry limestone cliff—**6**—V-13-2-17.

*Radula fulvifolia* (Hook. f. et Taylor) Gottsche, Lindenb. et Nees (= *Radula meyeri* Steph.)—1325—Partly shaded mesic cliff—**1—**V-1-22-17.

*Radula inouei* K. Yamada—2835, 2957—Open moist cliffs and mesic tree trunks—**29**, **31**—V-10-10-22, V-9-68-22—*Heteroscyphus tener*, *Plagiochila semidecurrens*.

* *Radula japonica* Gottsche ex Steph.—1557—Open moist boulder near stream—**5—**V-8-1-22.

*Radula javanica* Gottsche—1325, 1520, 2030, 2060, 2610—Open to partly shaded mesic to moist cliffs, tree trunks and branches—**1**, **5**, **13**, **17**, **18**—V-1-3-17, V-12-18-17, V-3-4-17, V-4-14-17—*Bazzania tridens*, *Drepanolejeunea angustifolia*, *Heteroscyphus tener*, *Lejeunea flava*, *Plicanthus birmensis*.

*Radula kojana* Steph.—2670—Partly shaded moist boulder near stream—**22**—V-19-12-18—*Jubula javanica*.

*Radula madagascariensis* Gottsche—1900, 2100, 2210, 2670—Open to partly shaded mesic tree trunks, branches and cliffs—**8**, **12**, **16**, **22**—V-1-55-16, V-2-117-16, V-19-18-18, V-5-44-17—*Bazzania pearsonii*, *Frullania yuennanensis*, *Heteroscyphus tene*r, *Plagiochila semidecurrens* [4].

*Riccardia decrescens* (Steph.) S. Hatt.—2210, 1520, 2727—Partly shaded moist to wet cliffs near stream—**5**, **16**, **19**—V-5-60-17, V-15-27-18, V-3-51-17—*Bazzania japonica*, *B. tridens*, *Heteroscyphus coalitus*, *Scapania maxima*, *Lobatiriccardia yunnanensis*.

*Riccardia flavovirens* Furuki—1410, 1557, 1700-1900—Open to partly shaded moist to wet cliffs, mostly near streams and waterfalls or on soil near forest ponds—**3**, **5**, **8**, **9**—V-1-113-16, V-2-27-17, V-8-29-22—*Heteroscyphus coalitus*, *Scapania undulata* [4].

* *Riccardia glauca* Furuki—1325—Open wet cliff—**1**—V-1-45-17.

* *Riccardia latifrondoides* Schiffn.—1520—Partly shaded mesic decaying wood—**5**—V-3-37-17.

*Riccardia nagasakiensis* (Steph.) S. Hatt.—1840, 2210, 2610, 2835—Open to partly shaded moist to wet cliffs near or in streambed—**8**, **16**, **21**, **31**—V-10-29-17, V-10-37-22, V-11-29-17, V-5-9-17—*Cephalozia conchata*, *Lepidozia vitrea*, *Plectocolea rosulans*.

*Riccardia palmata* (Hedw.) Carruth.—2610—Open moist cliff near stream—**21**—V-11-7-17—*Cephalozia bicuspidata*, *Delavayella serrata*.

* *Riccardia parvula* Schiffn.—1325, 1410, 2210, 2610—Partly shaded to open moist to wet cliffs and boulders, mostly near streams, also partly shaded moist decaying wood—**1**, **3**, **16**, **21**—V-1-48-17, V-11-31-17, V-2-10-17, V-5-16-17—*Calypogeia apiculata*, *C. granulata*, *Heteroscyphus argutus*, *H. coalitus*, *Lepidozia vitrea*, *Plagiochila sciophila*, *Plectocolea granulata*, *P. rosulans*, *P. tetragona*.

*Riccardia pumila* Furuki—1557, 1900, 2100—Open to partly shaded wet cliffs near streams and waterfalls, once partly shaded moist decaying wood—**5**, **8**, **12**—V-1-125-16, V-2-145-16, V-8-44-22—*Calypogeia cuspidata*, *Cephalozia conchata*, *Lepidozia omeiensis* [4].

*Riccardia pusilla* Grolle—1840—Partly shaded moist decaying wood—**8—**V-10-7-17.

*Saccogynidium muricellum* (De Not.) Grolle—1325, 1520, 1557—Open to partly shaded mesic to moist cliffs, including those near streams, boulders and clayish soil—**1**, **5**—V-1-24-17, V-3-36-17, V-8-9-22—*Calypogeia lunata*, *Heteroscyphus coalitus*, *Kurzia borneensis*, *Schiffneria hyalina*, *Schistochila doriae*.

*Scapania contorta* Mitt.—2030, 2610, 2900—Open to partly shaded moist cliffs, rarely wet cliffs in the streambed—**21**, **23**, **26**—V-8-37-17, V-9-33-17, V-11-27-17—*Bazzania ovistipula*, *Calypogeia aeruginosa*, *Herbertus armitanus*, *Lepidozia faurieana*, *Marsupella vietnamica*, *Mylia vietnamica*, *Scapania undulata*.

*Scapania ciliatospinosa* Horik.—2014, 2030, 2821, 2846, 2900, 2957, 3105, 3143—Open to partly shaded moist cliffs, rarely those near streams—**11**, **20**, **23**, **24**, **26**, **27**, **29**, **30**—V-3-22-16, V-16-5-18, V-17-23-18, V-18-18-18, V-21-14-18, V-8-10-17, V-9-11-17, V-9-16-22—*Anastrophyllum bidens*, *Bazzania angustistipula*, *B. ovistipula*, *Calypogeia aeruginosa*, *C. granulata*, *C. vietnamica*, *Cephaloziella willisana*, *Cololejeunea schmidtii*, *Cryptolophocolea sikkimensis*, *Delavayella serrata*, *Herbertus ramosus*, *Lepidozia subtransversa*, *Metacalypogeia alternifolia*, *Plagiochila hyalodermica*, *P. pseudofirma*, *P. semidecurrens*, *Radula cavifolia*, *Scapania metahimalayana*, *S. ornithopoides*, *Schistochilopsis setosa*, *Solenostoma faurieanum*, *S. heterolimbatum* [4].

* *Scapania ferruginea* (Lehm. et Lindenb.) Gottsche—2670, 2846, 2884, 2957—Open to partly shaded moist cliffs, including those near streams—**22**, **23**, **24**, **29**—V-17-43-18, V-19-43-18, V-20-9-18, V-9-26-22—*Aneura pinguis*, *Bazzania praerupta*, *Calypogeia aeruginosa*, *Cryptolophocolea sikkimensis*, *Herbertus ramosus*, *Metacalypogeia alternifolia*, *Plagiochila hyalodermica*, *P. semidecurrens*, *Scapania metahimalayana*, *Schistochilopsis setosa*, *Solenostoma faurieanum*.

*Scapania ligulata* Steph.—1900, 2030, 2100, 2210, 2610, 2900, 2957, 3143—Open to partly shaded moist to wet cliffs, including those near streams and in the streambeds—**8**, **12**, **16**, **18**, **21**, **23**, **29**, **30**—V-1-131-16, V-11-25-17, V-2-143-16, V-3-97-16, V-4-19-17, V-5-63-17, V-9-18-17, V-9-43-22—*Calypogeia granulata*, *Isotachis japonica*, *Marsupella stoloniformis*, *Metacalypogeia alternifolia*, *Scapania ornithopoides*, *Solenostoma suborbiculatum* [4].

*Scapania maxima* Horik.—1840, 1995, 2014, 2030, 2210, 2610, 2727—Open to partly shaded mesic to moist cliffs, including those near streams—**8**, **10**, **11**, **16**, **17**, **19**, **26**—V-10-18-17, V-12-4-17, V-15-27-18, V-21-12-18, V-5-56-17, V-7-25-17, V-8-27-17– *Bazzania japonica*, *Bazzania tridens*, *Calypogeia granulata*, *Heteroscyphus coalitus*, *Kurzia makinoana*, *Lepidozia faurieana*, *Metacalypogeia alternifolia*, *Plagiochila beddomei*, *Plectocolea rosulans*, *Riccardia decrescens*, *Trichocolea rudimentaris*.

*Scapania metahimalayana* Vilnet et Bakalin—2835, 2900, 2957—Open, rarely partly shaded, moist to wet cliffs, including those near streams—**23**, **29**, **31**—V-10-17-22, V-9-17a-17 (holotype), V-9-60-22—*Bazzania ovistipula*, *Calycularia crispula*, *Calypogeia aeruginosa*, *Kurzia makinoana*, *Metacalypogeia alternifolia*, *Plagiochila trabeculata*, *Scapania ciliatospinosa*, *S. ferruginea*, *Solenostoma faurieanum*, *S. suborbiculatum* [5].

*Scapania ornithopoides* (With.) Waddell—1995, 2014, 2030, 2210, 2610, 2727, 2821, 2846, 2900, 3105, 3143—Open, rarely partly shaded, moist to wet cliffs, including those near streams—**10**, **11**, **16**, **19**, **20**, **21**, **23**, **24**, **26**, **27**, **30—**V-3-13-16, V-11-8-17, V-15-41-18, V-16-26-18, V-17-11-18, V-18-18-18, V-21-18-18, V-5-63-17, V-7-26-17, V-8-10-17, V-9-11-17—*Anastrophyllum bidens*, *Aneura pinguis*, *Bazzania angustistipula*, *B. praerupta*, *Calypogeia granulata*, *C. lunata*, *C. vietnamica*, *Delavayella serrata*, *Fuscocephaloziopsis gollanii*, *Herbertus armitanus*, *Heteroscyphus coalitus*, *Kurzia makinoana*, *Lepidozia subtransversa*, *Metacalypogeia alternifolia*, *Mnioloma fuscum*, *Mylia vietnamica*, *Plagiochila pseudofirma*, *P. semidecurrens*, *Radula cavifolia*, *Scapania ciliatospinosa*, *S. ligulata*, *Schistochilopsis setosa*, *Solenostoma pseudocyclops* [4].

*Scapania parvitexta* Steph.—1520, 1995, 2100—Open to partly shaded moist to wet cliffs near streams—**5**, **10**, **12**—V-2-129-16, V-3-19-17, V-7-21-17—*Calypogeia apiculata* [4].

*Scapania pseudojavanica* Vilnet et Bakalin—1900, 1995, 2835—Open wet cliffs near streams, rarely mesic boulders in part shade—**8**, **10**, **31**—V-1-9-16, V-10-1-22, V-7-17-17– as *Scapania javanica* [4].

*Scapania undulata* (L.) Dumort.—1410, 1520, 1840, 2030, 2210, 2610—Open to partly shaded cliffs and boulders, mostly near streams, rarely open mesic cliffs—**3**, **5**, **8**, **16**, **17**, **18**, **21**, **26**—V-10-8-17, V-11-10-17, V-12-20-17, V-2-27-17, V-3-78-17, V-4-13-17, V-5-47-17, V-8-6-17—*Kurzia borneensis*, *Marsupella vietnamica*, *Riccardia flavovirens*, *Scapania contorta*.

*Schiffneria hyalina* Steph.—1520, 2060, 2610, 2670, 2727, 3143—Partly shaded mesic to moist and wet cliffs, boulders, humus on steep slope—**5**, **13**, **17**, **19**, **22**, **22**, **30**—V-3-101-16, V-12-12-17, V-15-1-18, V-19-15-18, V-3-49-17, V-6-28-17—*Calypogeia granulata*, *Fuscocephaloziopsis catenulata* subsp. *nipponica*, *F. gollanii*, *Heteroscyphus argutus*, *Radula cavifolia*, *Saccogynidium muricellum* [4].

*Schistochila aligera* (Nees et Blume) J.B. Jack et Steph.—Phan-si-pan, 2400 m a.s.l. [15].

* *Schistochila doriae* (De Not.) Trevis.—1325, 1520, 1840—Open to partly shaded mesic to moist cliffs and boulders, including those near streams—V-1-24-17, V-10-23-17, V-3-26-17—**1**, **5**, **8**—*Bazzania tridens*, *Heteroscyphus argutus*, *H. coalitus*, *Saccogynidium muricellum*.

*Schistochila sciurea* (Nees) Schiffn.—1900—Partly shaded mesic tree trunk base—V-1-49-16—**8** [4].

*Schistochilopsis setosa* (Mitt.) Konstant.—2030, 2610, 2670, 2727, 2846, 2900, 2957, 3143—Open to partly shaded mesic to moist cliffs, including those near streams—**19**, **21**, **22**, **23**, **24**, **26**, **29**, **30—**V-11-4-17, V-15-14-18, V-17-34-18, V-19-39-18, V-3-32-16, V-8-21-17, V-9-36-17, V-9-67-22—*Anastrophyllum bidens*, *Bazzania ovistipula*, *Bazzania praerupta*, *Cryptolophocolea sikkimensis*, *Fuscocephaloziopsis catenulata* subsp. *nipponica*, *F. gollanii*, *Herbertus armitanus*, *Kurzia makinoana*, *Lepidozia omeiensis*, *L. subtransversa*, *Metacalypogeia alternifolia*, *Mnioloma fuscum*, *Plagiochila gracilis*, *P. hyalodermica*, *P. semidecurrens*, *P. trabeculata*, *Scapania ciliatospinosa*, *S. ferruginea*, *S. ornithopoides*, *Solenostoma pseudocyclops* [4].

*Solenostoma appressifolium* var. *minus* (Amakawa) Váňa et D.G. Long—1520—Partly shaded mesic cliff—**5**—V-3-90-17—*Jackiella javanica*.

* *Solenostoma faurieanum* (Beauverd) R.M. Schust.—2835, 2957—Open, rarely partly shaded moist cliffs, including those near streams—**29**, **31**—V-10-27-22, V-9-31-22—*Calycularia crispula*, *Calypogeia aeruginosa*, *Cephalozia conchata*, *Marsupella vietnamica*, *Metacalypogeia alternifolia*, *Scapania ciliatospinosa*, *S. ferruginea*, *S. metahimalayana*, *Solenostoma parvitextum*.

*Solenostoma heterolimbatum* (Amakawa) Váňa et D.G.Long—2100—Partly shaded moist cliffs—V-2-108-16—**12** [4].

* *Solenostoma parvitextum* (Amakawa) Váňa et D.G. Long—2835, 2957—Open to partly shaded moist to wet cliffs, including those near streams—**29**, **31**—V-10-2-22, V-9-62-22—*Solenostoma faurieanum*.

* *Solenostoma pseudocyclops* (Inoue) Váňa et D.G. Long—2846—Open to partly shaded moist cliffs—**24**—V-17-15-18—*Scapania ornithopoides*, *Schistochilopsis setosa*.

* *Solenostoma rotundatum* Amakawa—1325, 1557– Open mesic clayish road cut, partly shaded moist boulder near stream—**1**, **5**—V-1-19-17, V-8-12a-22—*Calypogeia cuspidata*.

*Solenostoma schaulianum* (Steph.) Váňa et D.G. Long—2670—Partly shaded moist cliff near stream—**22**—V-19-21-18 [8].

*Solenostoma suborbiculatum* (Amakawa) Váňa et D.G. Long—2610, 2821, 2835, 2957—Open to partly shaded moist to wet cliffs, including those near streams and in the streambed—**20**, **21**, **29**, **31**—V-10-3-22, V-11-14-17, V-16-18-18, V-9-60-22—*Calypogeia aeruginosa*, *C. cuspidata*, *Jubula javanica*, *Marsupella stoloniformis*, *M. vietnamica*, *Scapania ligulata*, *S. metahimalayana*.

*Southbya grollei* N. Kitag.—1350—Open, rather dry limestone—V-4-32-16—**2** [4].

*Sphenolobopsis pearsonii* (Spruce) R.M. Schust.—2030, 2610, 2835, 2957—Open to partly shaded moist to wet cliffs, once on bamboo trunk at 1 m above the ground—V-10-15b-22, V-11-19-17, V-8-64-17, V-9-5-22—**21**, **26**, **29**, **31**—*Bazzania ovistipula*, *Calypogeia tosana*, *Marsupella stoloniformis*, *Radula cavifolia*, *Vietnamiella epiphytica* [8].

*Spruceanthus semirepandus* (Nees) Verd.—1565, 1620, 2030, 2210, 2610, 2821—Open to partly shaded mesic to moist tree trunk branches, decaying wood, rarely cliffs—**6**, **15**, **16**, **17**, **18**, **20**—V-11-17-22, V-12-19-17, V-13-18-17, V-16-2-18, V-4-38-17, V-5-25-17—*Bazzania ovistipula*, *Lejeunea flava*, *L. parva*, *Plagiochila semidecurrens*.

*Syzygiella autumnalis* (DC.) K. Feldberg, Váňa, Hentschel et Heinrichs—1995—Open moist cliff near stream—**10**—V-7-5-17—*Kurzia makinoana*.

*Syzygiella elongella* (Taylor) K. Feldberg, Váňa, Hentschel et Heinrichs—1840, 2060, 3105—Open mesic to moist tree trunks and branches, rarely moist cliffs on slopes—**8**, **13**, **27**—V-10-16-17, V-18-12-18, V-6-32-17—*Chiastocaulon fimbriatum*, *Cylindrocolea recurvifolia*, *Drepanolejeunea angustifolia*, *Heteroscyphus tener*, *Lopholejeunea subfusca*, *Plagiochila semidecurrens*, *Plicanthus birmensis*, *Radula cavifolia* [8].

*Syzygiella nipponica* (S. Hatt.) K. Feldberg, Váňa, Hentschel et Heinrichs—2846—Partly shaded moist cliff—**24**—V-17-12-18—*Anastrophyllum bidens*, *Bazzania praerupta*, *Scapania ornithopoides*.

*Thysananthus comosus* Lindenb.—Sapa, 1650 m a.s.l. [13].

*Trichocolea japonica* T. Katag.—1900—Partly shaded mesic decaying stump—**8**—V-1-26-16 [4].

*Trichocolea pluma* Dumort.—2060, 2670, 2727, 2884—Open to partly shaded moist boulders, including those near streams, humus on steep slopes—**13**, **19**, **22**, **23**—V-15-7-18, V-19-49-18, V-20-12-18, V-6-26-17—*Heteroscyphus coalitus*.

*Trichocolea rudimentaris* Steph.—2014—Open moist cliff near stream—**11**—V-21-12a-18—*Bazzania japonica*, *Scapania maxima* [8].

*Trichocolea tomentella* (Ehrh.) Dumort.—1840, 1900, 2014, 2030, 2060, 2100, 2610, 2670, 2727—Open, rarely partly shaded, moist cliffs and boulders, including those near streams—**8**, **11**, **13**, **19**, **21**, **22**, **26**, **30**—V-1-89-16, V-3-62-16, V-10-26-17, V-11-2-17, V-15-6-18, V-19-47-18, V-21-13-18, V-6-1-17, V-8-45-17—*Heteroscyphus coalitus* [4].

*Tuzibeanthus chinensis* (Steph.) Mizut.—1620—Open dry limestone cliff—**7—**V-13-1-17.

*Vietnamiella epiphytica* Bakalin et Vilnet—2835, 2846, 2884, 2900—Open, rarely partly shaded, mesic *Rhododendron* branches—**23**, **23**, **24**, **31—**V-10-12-22, V-17-26-18, V-20-6-18, V-9-6-17—*Frullania tagawana*, *Radula cavifolia*, *Sphenolobopsis pearsonii*.

*Wiesnerella denudata* (Mitt.) Steph.—1410, 1520, 1557—Open to partly shaded mesic to moist and wet cliffs, mostly those near stream—**3**, **5**—V-2-9-17, V-3-85-17, V-8-31-22—*Conocephalum japonicum* [8].

### 2.3. Altitudinal Trends in the Formation

**Figure 1:**
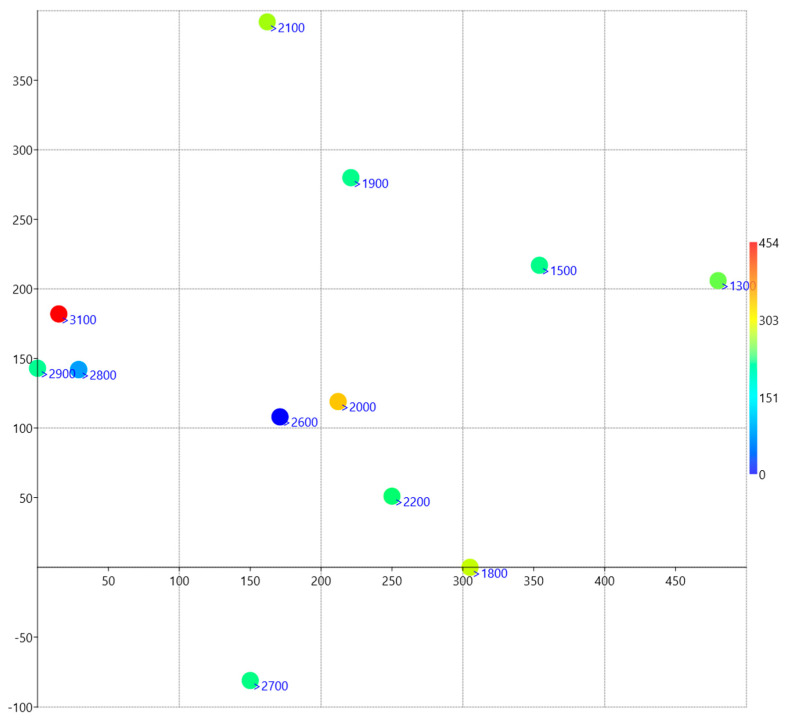
The distribution of the altitudinal floral fragments by 100−m gap on the DCA diagram (the third axis is the color gradient from deep blue to deep red).

The distribution of the altitudinal floral fragments by a 100-m gap on the DCA diagram (Figure 1) shows an almost even distribution of floras across the diagram. The visual proximity in Figure 1 of altitudinal belts >2800, >2900, >3100 is not actually real, as follows from the table of distances between floras of the mentioned altitudinal zones (Table 2 and Table 3) due to underestimations in third axis distances. In general, the value in the x-axis is almost clearly inversely proportional to the elevations: the floras are distributed from the highest to the lowest. According to the distance table (Table 3), the distances between floras gradually increase as the difference between the elevations increases. There are, however, deviations from this rule. For example, flora in the >2200 range is more closely related to the floras of the low altitudinal belts than even the lower-lying floras >2000 and >2100. The floral fragment >2200 is quite unique, and its relationship with the flora >1800 is generally the closest of all the range floras involved in the analysis. In general, compared to the average distances, distances above average are observed for altitude differences of more than 1000 m.

**Figure 2:**
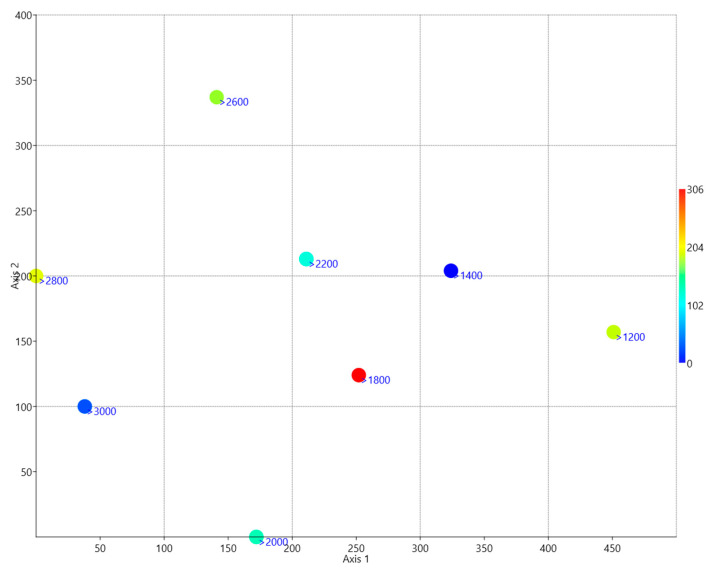
The distribution of the altitudinal floral fragments by 200-m gap on the DCA diagram (the third axis is the color gradient from deep blue to deep red).

The distribution of altitudinal floral fragments with 200-m gaps (Figure 2) generally shows the same characteristics. At the same time, due to the averaging effect, the average distances for this matrix (Table 4 and Table 5) are lower than the average distances between floras specified over 100-m segments. The highest distances (a hundred conventional units exceeding the distances between any other flora) are the distances between the lowest altitude level (>1200) and the highest (>2800, >3000) (Table 5).

### 2.4. Floristic Connections of Studied Area with Other Floras

**Figure 3:**
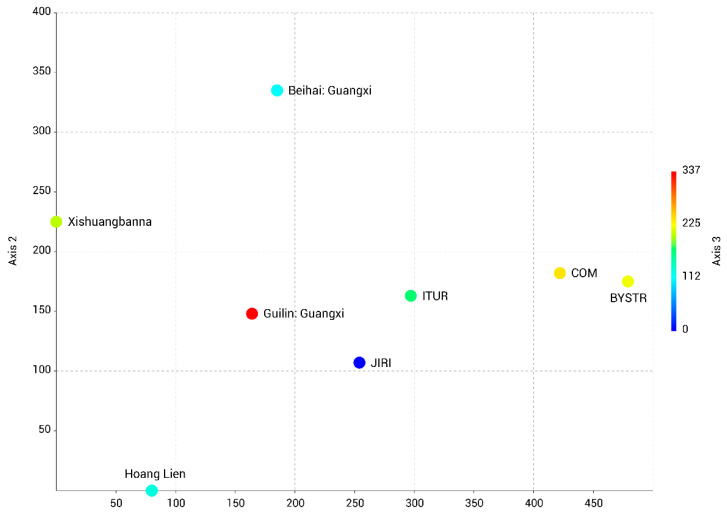
Comparison of the flora distribution in the DCA bubble chart (the third axis is the color gradient from deep blue to deep red).

The obtained DCA diagram (Figure 3) includes all eight floras: four territorially close at the southern tip of the East Asian region (including the studied area) and four from its northern tip and the middle part of Pacific Northeast Asia. Clear clusters are not distinguished on the diagram, and the position of the floras on the abscissa axis is directly proportional to the increasing latitude, although the distribution is not even and a significant difference in latitude may not be reflected in an equally significant difference in x-axis distance.

For each group of floras, distances in kilometers (Table 6 and Table 7) and distances in conventional units (Table 8 and Table 9) from the results of a three-dimensional distribution obtained by DCA were determined. Since all eight floras were included in one matrix, direct comparison of DCA distances in the two groups is possible.

The average distance in kilometers between the floras of the first group (Hoàng Liên plus) is 652 km. The average distance between the floras of the second group (Iturup plus) is 2122 km, which exceeds the average distance in the first group by three times. At the same time, the average distances between floras in conventional units in the first group is 271, while, in the second, it is only 200. Thus, despite larger distances in kilometers, the relationships between floras in the second group are closer. In the first group, above average distances are found between the Beihai and Hoàng Liên Sơn floras, although the distance, expressed in kilometers, is below average between them. In the second group, the above-average distance in conventional units is between the Jiri-san flora (the southernmost of those compared in the group) and two floras in Northeast Asia (Bystrinsky and Commanders), separated by more than 3000 km. At the same time, the Iturup flora, located at the northernmost tip of the East Asian region, turns out to be quite close to all other floras of the second group, despite serious geodesic distances varying from 1450 (Bystrinsky) to 2000 (Jiri-san) kilometers. Thus, there is higher disunity in floristic composition in selected floras in the south of the East Asian Floristic Region (Hoàng Liên plus) than in its northern extremity plus the middle part of Pacific Northeast Asia (Iturup plus).

## 3. Discussion

### 3.1. Taxonomic Diversity

The total known taxonomic diversity reaches 279 species and exceeds that in the compared floras of South China. Around North Indochina, this is presumably the richest known liverwort flora. Lejeuneaceae occupies the first position in the list of the top 10 families. However, it covers only 16.5% of the total flora. According to published reports for relatively well-studied and mountainous Malaysia [30], Lejeuneaceae in that distinctly tropical mountainous country comprises 40% of the total diversity. Even more so, the comparatively low value of Lejeuneaceae is evident in the generic spectrum, where *Lejeunea* ranks third and *Cololejeunea*, with eight species, only 10th. No additional Lejeuneaceae genera were included in the leading top families in Hoàng Liên Sơn. This can be explained by two factors: (1) the insufficient study of Lejeuneaceae and (2) its objectively lower value in the mountain flora, where the mountain species of the Sino-Himalayan distribution play a significant role in its formation (among which Lejeuneaceae are not numerous). Most likely, both factors act together, which also supports the assumption that the taxonomic diversity of species known in the flora will be further increased with future targeted research. The high proportion of Sino-Himalayan species in the flora is evidenced by the high values of the genera *Scapania* and *Calypogeia*, which are generally diverse in the mountains of the Holarctic but are especially diverse in the mountain mesophytic floras of the Sino-Himalayas [31]. The Sino-Himalayan influence on the formation of the flora of Northern Vietnam was noted earlier by our group [4,8]. This also led to the conclusion that the studied flora is, although rich in tropical elements, not tropical by the general content.

Twenty-six species are reported for the first time for the flora of Vietnam (marked with an asterisk in the list). Some of them, such as *Cephaloziella willisana*, *Jubula sikkimensis*, *Kurzia borneensis* and *Plagiochila hokinensis*, have a questionable taxonomic status. Together, all new records are to be expected, taking the general distribution patterns of all novelties into account. Most of them are widespread in the Sino-Himalayas. Additionally, it is worth mentioning that some new-for-Vietnam species of *Riccardia* (*R. glauca*, *R. latifrondoides*, *R. parvula*) and *Solenostoma* (*S. faurieanum*, *S. parvitextum*, *S. pseudocyclops*, S*. rotundatum*) may actually be incorrectly identified and represent weakly morphologically diversified new-for-science taxa and belong to species geographically vicarious in relation to those mentioned. In addition, the same problem is estimated to occur in almost all large genera of the studied flora, although not so prominently. To resolve this issue, it is necessary to carry out special molecular genetic studies, some of which we intend to conduct in the near future. In addition, it is worth mentioning that among the mentioned genera, as well as among others, there are a number of specimens identified to the genus only (and not included in the checklist) and presumably belonging to species that are new to science. Thus, the presented list is incomplete. The exact number of species that will be additionally found after further research in the study area is difficult to predict, but it is clear that new research will increase the number of known species to no less than 30–50 taxa.

### 3.2. Altitudinal Trends

The greatest liverwort diversity is observed within altitudinal one-hundredth meter diapasons of 1500–1599 m a.s.l. (104 species) and 2000–2099 m a.s.l. m. (105 species). This can be partly due to random factors—for example, the stochastically greater concentration of studied localities (and, as a result, more exhaustive collection of the flora) and more time spent on research. However, at the same time, it can be noted that both of these intervals are peculiar boundaries in the altitudinal distribution of a number of taxa. For example, the diapason 1500–1599 m a.s.l. is an upper limit for *Drepanolejeunea commutata*, *Lejeunea obscura*, *Lepidozia subintegra*, *Leptolejeunea elliptica*, *Mastigophora diclados*, *Plectocolea granulata*, *P. hasskarliana*, *P. tetragona*, *Saccogynidium muricellum*, etc., in the area treated. The same diapason is also the lower limit for *Aneura pinguis*, *Bazzania japonica*, *Calycularia crispula*, *Calypogeia cuspidata*, *C. granulata*, *C. lunata*, *C. tosana*, *Cephalozia hamatiloba*, *C. siamensis*, *Cylindrocolea recurvifolia*, *Frullania moniliata*, *Fuscocephaloziopsis gollanii*, etc. In addition, most of the studied limestones are concentrated in this diapason, which leads to the enrichment of the flora with basiphilic species, such as *Plagiochasma cordatum*.

The diapason of 2000–2099 m a.s.l. m. in this respect is even more remarkable. This is the lower limit for the distribution of a number of mountain subtropical species, such as *Anastrophyllum bidens*, *Bazzania praerupta*, *Calypogeia pseudocuspidata*, *C. sinensis*, *Cephaloziella willisana*, *Cryptolophocolea sikkimensis*, *Delavayella serrata*, *Fuscocephaloziopsis catenulata* ssp. *nipponica*, *Herbertus armitanus*, *H. dicranus*, *Lejeunea parva*, *Plagiochila durelii*, *P. hyalodermica*, etc. The same diapason is the upper limit for *Cololejeunea hasskarliana*, *Drepanolejeunea angustifolia*, *Lejeunea neelgherriana*, *L. subacuta*, *Lopholejeunea nigricans*, *L. subfusca*, etc. A number of species not found in other altitude ranges were found in this belt: *Asterella cruciata*, *Bazzania angustifolia*, *B. faurieana*, etc.

In fact, there are more species that interrupt their distribution in both diapasons, but given the unevenness of knowledge (since the primary task was to identify the general diversity and not the patterns of altitudinal distribution), it is hardly possible to carry out any statistically reliable calculations on this issue.

### 3.3. Relationships and Diversification with Adjacent Areas

As noted in the Materials and Methods and Results sections, the comparison with other floras has an evidently “one-sided” nature, since the studied flora is the southernmost of those compared. This is determined by the lack of representative data for the comparison of local floras in Northern Indochina. However, considering the proportion of families in the total spectrum and, especially, the generic spectrum of the studied flora, we suggest that the studied flora is also predominantly mountainous subtropical, and such a comparison seems to be possible (although certainly not ideal). A comparison of four selected floras at the southern flank of the East Asian Floristic Region showed that, despite the relatively small kilometric distances between the compared floras, the interrelationships between them are rather low. Moreover, the relationships are smaller than the relationships between the floras situated at much greater distances (in kilometers) at the northern tip of the East Asian region and even in Pacific Northeast Asia. This feature of the strong disunity of floras in the Sino-Himalayas was revealed long ago (although it has a very indirect relation to the area covered by the compared floras) and was described with the possible main trends approximately 100 years ago [32,33,34]. The fundamental difference between the mountain floras of the Alps and the Sino-Himalayas was emphasized at the same time. Ward ([34]: 73–74) wrote, “It is necessary to remember that this Himalayan-Chinese flora is not alpine in the European sense. The Alps of Europe are generally supposed to have derived some of their flora from the Arctic, but clearly the Himalaya did not, and the resemblance between the European alpine flora and the Himalayan alpine flora is only a broad resemblance, despite the fact that the mountain ranges belong to one system”. The ancient features of the Sino-Himalayan flora, the absence of ice sheets in the foreseeable geological past and the close junction of the upper reaches of completely oppositely directed large rivers in the region led, so to speak, to the accumulation of uniqueness, which is largely (although far from that observed in glaciated Europe) deprived in more northerly Pacific Asian floras, even within the East Asian Floristic Region.

## 4. Materials and Methods

### 4.1. Area Treated Identification

**Figure 4:**
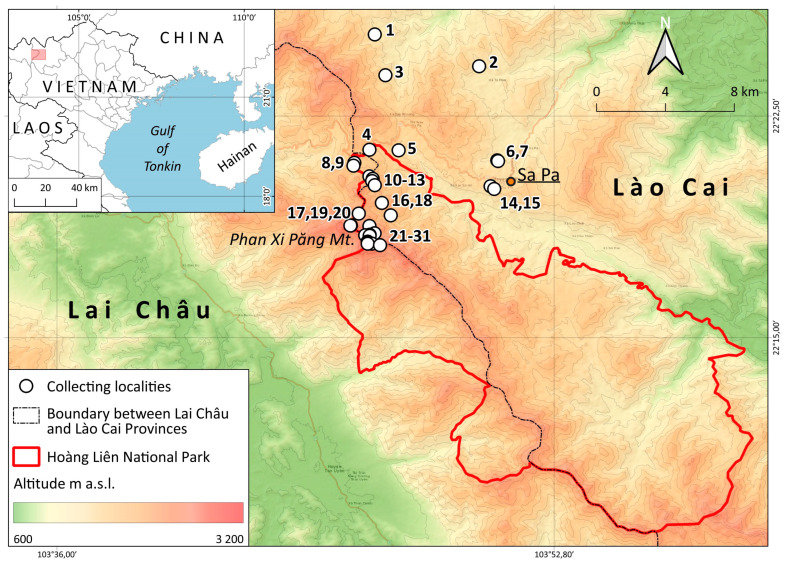
Collecting localities in Hoàng Liên Sơn Range in 2016, 2017, 2018 and 2022 corresponding to Table 4.

The study area is located in the central part of the Hoàng Liên Sơn Range, which extends from northwest to southeast for ca. 180 km, with a maximum width with side spurs reaching approximately 30 km. The area includes the highest mountain of Indochina—Phan Xi Pang, with an elevation of 3143 m a.s.l. in its summit. The elevation in the area treated starts at approximately 1300 m a.s.l. and reaches the Phan Xi Pang Peak. The national park itself has an area of 685 km^2^ (https://en.wikipedia.org/wiki/Ho%C3%A0ng_Li%C3%AAn_National_Park (accessed on 23 February 2023)) and lies within two Vietnamese provinces: Lao Cai and Lai Chau. It is worth mentioning that the study area (1) covers only a small portion of the park and (2) includes some adjacent areas that do not formally belong to the park. However, since this entire area is a single unit located on the macroslopes of the Phan Xi Pang Mt. and its adjacent spurs, there is no reason to divide the nationally protected area from those reserved on the provincial level and the non-reserved areas. Moreover, one of the most important liverwort diversity concentration points is observed in the Ham Rong municipal park located in Sapa Town. All collecting localities are listed in Table 10 and Figure 4 and distributed between 22.421° N and 22.302° N latitudinally and 103.767° E and 103.848° E longitudinally.

### 4.2. Natural Environments

Although, topographically, the Hoàng Liên Sơn Range is located on the Indochina Peninsula, geomorphologically, it has no association with the Indochina Block, which occupies the main area of Indochina. The main events that led to the formation of the Hoàng Liên Sơn Range occurred approximately 40–30 million years ago, when the collision of the Indian plate with the Asian continent caused the clockwise rotation of the Indochinese platform and “pressed” it into the South China Block [35,36]. The latter contributed to (1) the formation of folding, which is orographically expressed as a system of mountain ranges in Northwestern Vietnam, and (2) the rise of marine limestone deposits to heights reaching 2000 m a.s.l. and the opening of ancient granites and similar acidic rocks at altitudes exceeding 1500 m a.s.l. and now reaching more than 3000 m a.s.l. in Northern Indochina, with the highest point being Phan Xi Pang Mt. (3143 m a.s.l.). The formed mountain system is adjacent to the (1) Annam Mountains, located along the modern northeastern edge of the Indochina Block; (2) mountains of the Simao Block westward of the Hoàng Liên Sơn Range; and (3) the Hengduan Mountains, extending from the southeastern edge of Tibet (in the southern part, formed for the same reasons as in the mountain system of Northeastern Vietnam and described above) and contacting the Hoàng Liên Sơn Range in the northern tip of the latter [37]. Averyanov et al. ([3]: 34) wrote about this area: “Mountain systems here are generally composed of silicate rocks, mainly granite, gneiss, rhyolite or quartzite, formed as extensive magmatic intrusions of late Paleozoic and Mesozoic ages. Tertiary tectonic movements uplifted these montane terrains to modern elevations, and further erosion formed the present characteristic rocky landscape of this highland area”.

Therefore, geomorphologically and phytogeographically, the mountains of the northwestern tip of Vietnam are sound continuations of the Hengduan Mountains, through which a huge number of Sino-Himalayan flora elements penetrated into Indochina. The latter, in fact, makes this region the southern outpost of the East Asian subtropical flora [1,2]. At the same time, a mosaic combination of ranges with valleys occupied by tropical vegetation in lowlands also enriches the mountain systems of Northern Vietnam with tropical species. Another factor affecting the taxonomic diversity of the Hoàng Liên Sơn Range is the presence of a wide range of chemically different rocks, for instance, evidenced by pronounced outcrops of alkaline limestones (of karst origin) and acidic granites. Finally, a great altitudinal range, with the highest exact mountain in Indochina, Phan Xi Pang, allows the development in the upper belts of special types of vegetation not represented or poorly developed in other mountain systems, even in the northern part of Vietnam. Some authors [38] generally consider the vegetation of the upper belt of the Hoàng Liên Sơn Range to be temperate, instead of mountain subtropical. The latter statement may be questioned; however, the distribution, apparently relict, as in the case of *Abies delavayi* Franch., indicates the presence of temperate species in the Hoàng Liên Sơn Range. Moreover, several instances of climate cooling, including the Miocene cold interval (ca. 7–5.4 Ma), are very noticeable [39] and should influence the deep penetration of the oro-temperate flora into Northern Vietnam. Averyanov et al. ([3]: 29) stressed the temperate features of these highland communities: “A coniferous community of distinctly temperate affinities occurs in northern Vietnam at higher elevations between 2400–2900 m in only the northwestern part of the country, with occasional dominance of *Tsuga dumosa* and *Abies delavayi*”. The presumable relict occurrence of *Gymnomitrion rubidum* (Mitt.) Váňa, Crand.-Stotl. & Stotler, in the scattered community of *Abies delavayi* probably evidences that the temperate taxa that survived together (as a suite) are now alien to them, not temperate communities [9]. All of the above make the taxonomic diversity of the Hoàng Liên Sơn Range potentially high, even in comparison with other mountain systems. The earlier statistical analysis of the distribution of new records in Northern Vietnam [8] showed that the discovery of liverwort species new to the country is most likely in the upper mountain belts. The wide occurrence of Sino-Himalayan taxa in the upper belts is the most interesting and distinctive trait of the studied area.

However, in the general zonal characteristic, the dominant type of vegetation in the study area is evergreen montane and highland forests on silicate rocks at 1000–3000 m a.s.l [3]. In reality, the organization of floristic complexes is more complicated since communities are also developed on alkaline substrates. Moreover, altitudinal climatic differentiation supplemented the difference in substrates. These factors result in ([3]: 26) “a relatively sharp distinction in vegetation structure between lowland and montane forest communities. This delineation is marked by a decline in the number of taxa of tropical families such as the Anacardiaceae, Dipterocarpaceae, Euphorbiaceae, Meliaceae and Simaroubaceae and an increase in dominance of subtropical and temperate families like Fagaceae, Theaceae, Magnoliaceae and a diverse assemblage of conifers”.

**Figure 5:**
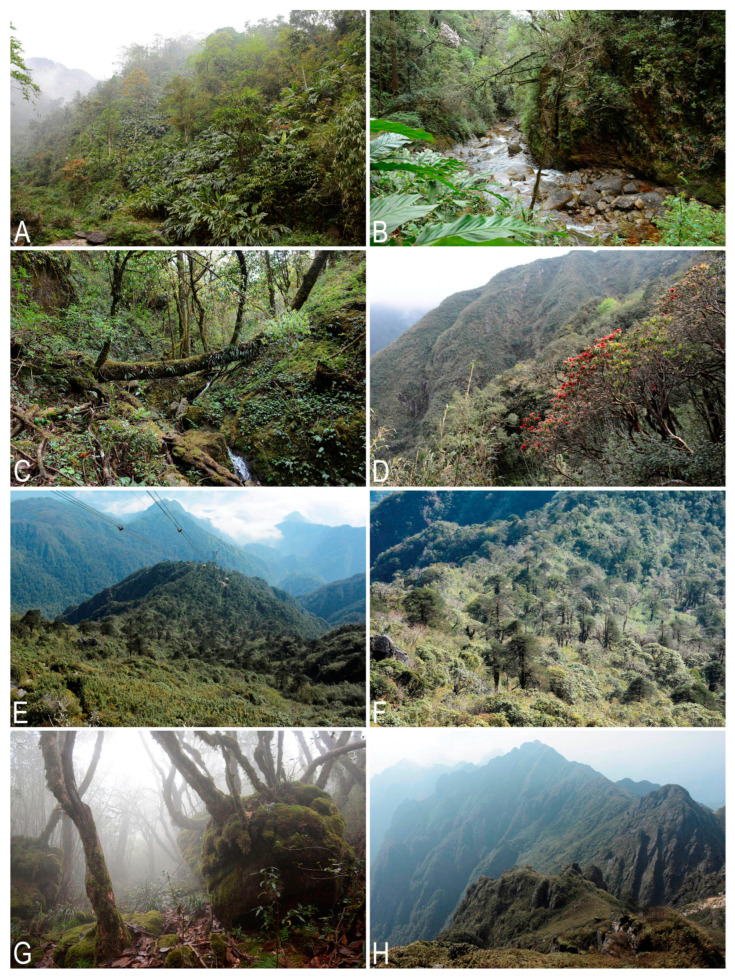
Landscapes and liverwort habitats in Hoàng Liên National Park and the adjacent areas: (**A**) evergreen south subtropical mountain forest in stream valley, 1325 m a.s.l., loc. 3; (**B**) evergreen south subtropical mountain forest in deep valley, northeast foot of Phan Xi Pang Mt., 2045 m a.s.l., loc. 18; (**C**) evergreen south subtropical mountain forest, 2210 m a.s.l.; (**D**) the ridge area of the range, where *Rhododendron tanastylum* Balf. f. & Kingdon-Ward is in the foreground, 2745 m a.s.l.; (**E**,**F**) view of the scattered community of *Abies delavayi* Franch. on the northeast slope of Phan Xi Pang Mt., 2670 m a.s.l., loc. 22 is within; (**G**) *Rhododendron* dominated forest with bamboo thickets and rocky outcrops, northeast slope of Phan Xi Pang Mt., 2962 m a.s.l., loc. 23; (**H**) the apical area of Phan Xi Pang Mt., view of the neighboring peak (2900 m) to the northwest from the Phan Xi Pang Mt., 3114 m a.s.l. (Photos by K.G. Klimova, 2017, 2018).

Due to elevations lower than 1300 m a.s.l. being absent in the studied area, we did not study the typical tropical forests developed in the lowlands of Northern Vietnam at elevations below 1000 m a.s.l. The typical landscapes and liverwort habitats are in Figure 5.

Since the studied area has mountainous relief, the climate (especially temperature) changes drastically with altitude and slope exposure. To determine the most general characteristics of the alternation of weather elements, we used the climate chart on the ClimateData website (https://en.climate-data.org/asia/vietnam/lao-cai-province/sa-pa-36229/ (accessed on 22 February 2023)) for the weather station located in Sapa Town. The station is situated at an altitude of 1489 m a.s.l. and provides features of the climate of the lower altitude level and its seasonality throughout the year in the area treated. According to the Köppen–Geiger climate classification, the climate in Sapa is temperate with warm summers and no dry season (Cfb according to the mentioned classification) [40]. Despite the term “no dry season”, Sapa has significant variability in the amount of rainfall that occurs during the seasons. In addition, temperature seasonality is quite pronounced. Although Sapa is located south of the Tropic of Cancer, the alternation of temperatures over the seasons is very well expressed here. The warmest quarter of the year is June–August. This period is also the wettest. The driest quarter of the year is from November to January, and the coldest quarter is observed with a slight shift—from December to February. The warmest month is July, and the coldest month is January.

Since the coordinates were known for all our collection localities, we then obtained the bioclimates from the WorldClim database (https://www.worldclim.org/data/bioclim.html (accessed on 22 February 2023)) for each point, which made it possible to compile a general characteristic for the study area. These data are placed in Table S1 and are discussed below. To characterize each point, all 19 bioclimates were obtained, which can be determined with a maximum accuracy of 30”, and if the points are closer, then the parameters could merge. Despite the inevitable averaging of the data (for example, all six highest points above 2900 m a.s.l. were combined into one cluster, and points 19 and 26, located on a steep slope, clearly show the climatic values of the upper altitude levels), the results indicate serious altitudinal differences in local climates across the altitudinal gradient. The lowest point is located at an altitude of 1325 m a.s.l., and the highest is located at an altitude of 3143 m a.s.l. The average annual temperature on this 1800-m diapason, when climbing into the mountains, gradually drops from almost 17 to 9 °C. The average maximum temperatures vary from 26 to 19.9 °C, and the average minimum temperatures range from 6.3 to –0.7 °C. The average temperature of the wettest quarter (coinciding with the warmest) varies from 21.8 to 14.0 °C, and the average temperature of the coldest quarter (almost the same as the driest) varies from 10.8 to 3.6 °C. The annual amount of precipitation, contrary to our expectations, does not increase when climbing the mountains and falls from 2335 to 1822 mm per year. Moreover, this change occurs mainly due to a decrease in the amount of precipitation in the warmest season. The amount of precipitation for the warmest quarter falls from 1254 to 998 mm depending on the elevation. The maximum amount of precipitation is observed in the diapason from 1500 to 1600 m a.s.l., thus not in the lowest localities. The precipitation in the driest quarter varies slightly, from 53 mm at the lowest levels to 35 mm at levels of 2000–2600 m a.s.l. m., and then slowly increasing to 39–40 mm in the apical part. Thus, the phenomenon known as the interception of moisture from moisture-bearing air masses is not observed here. At the same time, despite the decrease in the amount of precipitation toward the peaks, the vegetation of the upper belts does not have the traits of greater xerophyticity compared to the lower levels. This may be due to two factors: (1) a decrease in average temperatures at the upper levels, which also reduces the potential evaporation from the substrates and plants, and (2) frequent cloudiness in the near-top areas, which is not recorded as precipitation but significantly moistens the vegetation and reduces evaporation. One of the confirmations of this phenomenon is the wide distribution of epiphytic bryophytes, starting from 2200–2400 m a.s.l. The latter forms the appearance of communities resembling mossy forests (although not genetically related to them).

### 4.3. Literature Background and Specimen Collection

As is easily expected, the peculiar position and geological and topographic features of the Hoàng Liên Sơn Range have attracted the attention of many biologists, including botanists who have collected specimens on the range. The results of these studies are reflected in a number of published papers [13,14,15,16,17]. At the same time, it is worth noting that apart from our research group, only one hepatologist (Tamás Pócs) has worked on the Hoàng Liên Sơn Range. Moreover, he did not visit localities above 2000 m a.s.l. We were the first bryologists to visit the apical areas of the range (within area treated in the present account) in 2016, and since then have visited more than once. A number of new findings in the Vietnamese flora of liverworts from the Hoàng Liên Sơn Range were published earlier [4,5,6,7,8,9,10,11,12], although a total checklist of liverworts for the Hoàng Liên Sơn Range has never been published.

The territory of the park and adjacent areas, as identified in the present account, was surveyed by our group in 2016, 2017, 2018 and 2022, no more than a week each year, usually in April. A total of 1320 specimens were collected, with 2219 determinations, because specimens commonly have more than one species inside. All liverwort specimens after collection were stored in the fridge under anabiosis conditions to keep them alive and, therefore, prevent the destruction of oil bodies in the cells. In 2016, 2017 and 2018, the specimens were transported to Vladivostok to the Cryptogamic Biota Laboratory (Botanical Garden Institute, herbarium acronym VBGI) for identification; approximately 30% of these specimens were then frozen, and the “shock” nature of freezing led to the death of oil bodies in approximately 50% of the frozen material. In 2022, to avoid alive specimen death, all material was delivered to Hanoi to the Laboratory of Plant Ecology (Institute of Ecology and Biological Resources, herbarium acronym NH), where the material was identified by morphological methods. In both cases, the general appearance of plants and oil bodies was photographed for approximately 70% of the collections of 2016–2018 and 70% of the collection of 2022. Only after the end of the identification were all specimens dried. To take photographs of the collected plants, we used microscopes in the laboratories of VBGI and HN, with the most valuable ones in the Laboratory of Plant Ecology at the Institute of Ecology and Biological Resources of Vietnam Academy of Science and Technologies, including a Nikon SZM800M and Olympus BX43, both equipped with digital cameras.

The results of the identifications were later input into a database, from which an extraction was then obtained and later processed manually using PC software. Literary references were added to the obtained draft list. In most cases, it was impossible to reliably understand from old literary sources whether the distribution of the species was indicated within the park (the park was founded only in 2006) or belonged to its environs because, in the vast majority of cases, only the geographical description “Sapa” was indicated. Fortunately, in most cases, the literature provides data on the elevation above sea level of the collection locality. Presumably, all localities above 2000 m a.s.l. belong to the park, and those below 1700 are outside the park. However, the specimens collected between 1700 and 2000 m a.s.l. could be gathered both within the national park and outside it.

### 4.4. Flora Analysis Methods

Detrended correspondence analysis (DCA) was used to identify the trends in altitudinal formation of the studied flora and the relationships of the total taxonomic list of the studied flora with other flora lists for some adjacent areas. This multivariate statistical technique was chosen because it is suitable for finding the main factors or gradients in large, species-rich but usually sparse data matrices. DCA was performed using Past ver. 4.03c [41].

This type of analysis was applied to two issues raised.

(1) Comparison with the floras of adjacent areas. For comparison, we chose the richest local floras in the adjacent regions of Yunnan Province and the Guangxi Zhuang Autonomous Region in China. Unfortunately, there are no summarized data on local floras in Northern Thailand, and they are not in Laos, where all known diversity is limited by 66 species [18]. Thus, the comparison turned out to be rather one-sided, since it included data only on the floras of the areas adjacent to the north. A list of floras with indications of sources is given in Table 11.

In addition, since the distances between the coordinates of the positions of floras in a three-dimensional system are a dimensionless (conventional) value, we made an attempt to compare the average distances at the southern tip of the East Asian region with the average distance between the additionally involved floras at the northern tip of the East Asian Floristic Region and adjacent Northeast Asia, belonging to the Circumboreal Floristic Region [2]. Unfortunately, only a small number of local floras can be considered sufficiently studied. As a result, four additional floras were chosen, two of which are located in the north of the East Asian region, and two are in the middle part of Pacific Northeast Asia. These floras were Iturup Island, Jiri-san National Park, Bystrinsky Nature Park and the Commander Islands. The information about all compared floras is placed in Table 11.

Thus, a matrix was compiled, including a cumulative list of species for all 8 floras, in which the presence of a species was indicated by the number 1 and the absence by the sign 0. This matrix is provided in the Supplementary Material (Table S2). A summary diagram was made for all floras, and conditional coordinates were determined in a three-dimensional grid obtained by DCA for each set of floras mentioned above. Next, the distances were identified in conventional units between all floras of each set. In addition, the distances in kilometers along the geodesic line between all floras of each set (based on the central point of the compared floras) were determined for subsequent comparison of the comparability of the geographical distance with the distances in the DCA matrix.

(2) To formalize the description of changes in the liverwort flora of the studied area depending on elevation, two matrices were compiled on the distribution of taxa within the altitudinal range. Both matrices are based on the list of species of the study area, and the presence of a species within the altitudinal range is marked with a 1 (absence is 0). One matrix was compiled according to the distribution of taxa within the hundred-meter ranges (1300–1399, 1400–1499 m a.s.l., etc.), while the second operated on 200-m ranges. In the first case, the diapasons are named in formats such as >1300, >1400, etc., whicih means the gaps 1300–1399, 1400–1499, etc., correspondingly. In the second case, a gap >1200 means that the diapason is 1200–1399 m a.s.l. Because the ranges were unevenly explored, and, for some, too little data were collected, before being included in the analysis, all ranges in which less than 40 species were known were removed from the analysis to exclude aberrations due to insufficient data. The following ranges were excluded: 1400–1499, 1600–1699, 1700–1799, 2300–2399, 2400–2499, 2500–2599, 3000–3099, 1600–1799 and 2400–2599 (Table S3).

## 5. Conclusions

The liverwort flora of the studied area, located in the central part of the Hoàng Liên Sơn Range, is very rich taxonomically. The data presented in this paper will undoubtedly be supplemented in future targeted research. In general, the flora possesses distinct Sino-Himalayan features, which is a typical trait of the floras in the northern tip of Indochina. At the same time, a comparison with the nearest well-studied regional floras in Southern China showed the significant differentiation of the flora of the studied region from the nearest floras adjacent to the north. The uniqueness of the studied area is determined by the close interpenetration of the Sino-Himalayan flora into the Indochinese, which, due to the significant altitudinal range of the study area, contributed to the formation of an original and very rich flora that promises numerous new records in the future.

## Figures and Tables

**Table 1:** The leading families and genera in the studied flora.

Family	Number of Species	% of Total Number of Species in the Park	Genera	Number of Species	% of Total Number of Species in the Park
Lejeuneaceae	46	16.49%	*Plagiochila*	30	10.75%
Plagiochilaceae	33	11.83%	*Bazzania*	14	5.02%
Lepidoziaceae	26	9.32%	*Lejeunea*	13	4.66%
Solenostomataceae	15	5.38%	*Frullania*	12	4.30%
Scapaniaceae	15	5.38%	*Radula*	11	3.94%
Calypogeiaceae	13	4.66%	*Porella*	10	3.58%
Aneuraceae	12	4.30%	*Calypogeia*	10	3.58%
Frullaniaceae	12	4.30%	*Scapania*	10	3.58%
Porellaceae	10	3.58%	*Riccardia*	9	3.23%
Radulaceae	11	3.94%	*Cololejeunea*	8	2.87%
Total	193	69.18%	10	127	45.52%

**Table 2:** Normalized values of DCA for each compared 100-m altitudinal zone.

Altitudinal Zone	Axis 1	Axis 2	Axis 3
>1300	480	206	249
>1500	354	217	218
>1800	305	0	278
>1900	221	280	217
>2000	212	119	338
>2100	162	392	266
>2200	250	51	231
>2600	171	108	0
>2700	150	-81	220
>2800	29	142	93
>2900	0	143	213
>3100	15	182	454

**Table 3:** Distances between floras in a three-dimensional grid of coordinates, calculated for 100-m segments (average 296.72).

Zone	Distances											
>1300												
>1500	130.22											
>1800	271.85	230.41										
>1900	271.26	147.17	298.63									
>2000	295.49	210.16	162.51	201.60								
>2100	368.79	264.18	417.44	135.74	286.73							
>2200	277.94	196.32	88.52	231.25	132.35	353.91						
>2600	408.76	304.79	326.96	281.38	340.66	389.22	250.70					
>2700	438.30	361.14	184.26	367.93	240.35	475.38	165.97	290.80				
>2800	481.49	356.20	361.34	266.99	306.66	331.84	275.98	173.12	283.72			
>2900	485.45	361.69	343.07	260.05	247.28	301.75	267.00	275.38	269.68	123.46		
>3100	508.75	414.54	384.97	328.95	237.14	317.89	349.45	485.72	377.03	363.48	244.60	
	>1300	>1500	>1800	>1900	>2000	>2100	>2200	>2600	>2700	>2800	>2900	>3100

**Table 4:** Normalized values of DCA for each compared 200-m altitudinal zone.

Altitudinal Zone	Axis 1	Axis 2	Axis 3
>1200	451	157	187
>1400	324	204	0
>1800	252	124	306
>2000	172	0	131
>2200	211	213	116
>2600	141	337	176
>2800	0	200	196
>3000	38	100	32

**Table 5:** Distances between floras in a three-dimensional grid of coordinates, calculated for 200-m segments (average 275.50).

Zone	Distances							
>1200								
>1400	230.88							
>1800	234.20	324.38						
>2000	325.00	286.15	228.91					
>2200	256.47	162.19	213.78	217.06				
>2600	358.64	286.63	273.11	341.40	154.52			
>2800	453.13	378.69	285.27	271.68	226.03	197.61		
>3000	444.80	306.00	348.49	194.31	223.06	295.83	195.81	
	>1200	>1400	>1800	>2000	>2200	>2600	>2800	>3000

**Table 6:** Distances between the compared floras of the southern tip of the East Asian Floristic Region, km.

Name of Flora	Approximate Coordinates	Hoàng Liên	Xishuangbanna	Beihai: Guangxi
Hoàng Liên	22°17′ N 103°49′ E			
Xishuangbanna	21°59′ N 100°47′ E	320		
Beihai: Guangxi	21°41′ N 109° 05′ E	550	850	
Guilin: Guangxi	25°17′ N 110°18′ E	740	1035	420

**Table 7:** Distances between the compared floras of the northern tip of the East Asian Floristic Region and the middle part of Northeast Asia, km.

Abbreviation of Flora	Approximate Coordinates	ITUR	JIRI	BYSTR
ITUR	45°03′ N 147°46′ E			
JIRI	35°20′ N 127°43′ E	2000		
BYSTR	56°20′ N 158°28′ E	1450	3290	
COM	54°53′ N 166°56′ E	1750	3690	550

**Table 8:** Distances between the compared floras of the southern tip of the East Asian Floristic Region, in conventional units based on DCA analysis (average 270.57).

Name of Flora	Approximate Coordinates	Hoàng Liên	Xishuangbanna	Beihai: Guangxi
Hoàng Liên	22°17′ N 103°49′ E			
Xishuangbanna	21°59′ N 100°47′ E	251.215047		
Beihai: Guangxi	21°41′ N 109° 05′ E	351.274821	233.291663	
Guilin: Guangxi	25°17′ N 110°18′ E	271.073791	224.753198	291.78588

**Table 9:** Distances between the compared floras of the northern extremity of the East Asian Floristic Region and the middle part of Northeast Asia, in arbitrary units based on the DCA analysis.

Abbreviation of Flora	Approximate Coordinates	ITUR	JIRI	BYSTR
ITUR	45°03′ N 147°46′ E			
JIRI	35°20′ N 127°43′ E	185.002703		
BYSTR	56°20′ N 158°28′ E	188.862384	321.945648	
COM	54°53′ N 166°56′ E	142.625383	300.029999	59.8915687

**Table 10:** The geographic positions of collecting localities within studied area.

Number	Latitude, N	Longitude, E	Altitude, m a.s.l.	Number	Latitude, N	Longitude, E	Altitude, m a.s.l.
1	22.421	103.779	1325	17	22.32	103.77	2610
2	22.40305556	103.8377778	1350	18	22.319	103.788	2030
3	22.398	103.785	1410	19	22.31333333	103.7655556	2030
4	22.356	103.776	1800	20	22.31325	103.7652778	2821
5	22.35566667	103.7924278	1557	21	22.313	103.776	2610
6	22.35	103.848	1620	22	22.30888889	103.7788889	2670
7	22.34961	103.84842	1620	23	22.30805556	103.7761111	2900
8	22.34861111	103.7675	1840	24	22.30777778	103.7736111	2846
9	22.347	103.767	1900	25	22.3075	103.7761111	2900
10	22.341	103.776	1995	26	22.30416667	103.775	2030
11	22.33961111	103.7778333	2014	27	22.30361111	103.7755556	3105
12	22.33833333	103.7775	2610	28	22.30305556	103.7752778	3143
13	22.336	103.779	2060	29	22.30304167	103.7773694	2957
14	22.33542	103.84422	1521	30	22.303	103.775	3143
15	22.333875	103.846275	1565	31	22.30219167	103.7819667	2835
16	22.326	103.783	2210				

**Table 11:** The list of local flora involved in the comparison.

No.	Name/Abbreviation of Flora	Explanation of the Abbreviation, Literature Sources	Approximate Coordinates
1	Hoàng Liên	Hoàng Liên National Park and adjacent areas, North Vietnam [4,5,6,7,8,9,10,11,12], present account	56°00′ N 158°30′ E
2	Xishuangbanna	Xishuangbanna Dai Autonomous Prefecture, Yunnan Province, China [42]	21°59′ N 100°47′ E
3	Beihai: Guangxi	Beihai District, Guangxi Zhuang Autonomous Region, China [43]	21°41′ N 109° 05′ E
4	Guilin: Guangxi	Guilin District, Guangxi Zhuang Autonomous Region, China [43]	25°17′ N 110°18′ E
5	ITUR	Iturup Island, Kuril Islands [44,45]	45°00′ N 149°00′ E
6	JIRI	Jirisan Mts., Jirisan National Park, southern part of Korean Peninsula [46]	35°20′ N 127°40′ E
7	BYSTR	Bystrinsky Nature Park, Sredinnyi Range, Central Kamchatka [47]	56°00′ N 158°30′ E
8	COM	Commander Islands [48,49]	55°00′ N 166°00′ E

## Data Availability

Not applicable.

## Supplementary Materials

The following supporting information can be downloaded at: https://www.mdpi.com/article/10.3390/plants12091841/s1, Table S1: Obtained bioclimatic data for collecting localities; Table S2: DCA matrix of taxa distribution in compared floras; Table S3: DCA matrix of taxa distribution within the 100-m and 200-m altitudinal ranges.
